# Evolutionary emergence of infectious diseases in heterogeneous host populations

**DOI:** 10.1371/journal.pbio.2006738

**Published:** 2018-09-24

**Authors:** Hélène Chabas, Sébastien Lion, Antoine Nicot, Sean Meaden, Stineke van Houte, Sylvain Moineau, Lindi M. Wahl, Edze R. Westra, Sylvain Gandon

**Affiliations:** 1 CEFE UMR 5175, CNRS - Université de Montpellier - Université Paul-Valéry Montpellier – EPHE, Montpellier, France; 2 ESI and CEC, Biosciences, University of Exeter, Cornwall Campus, Penryn, United Kingdom; 3 Département de biochimie, microbiologie et de bio-informatique, Faculté des sciences et de génie, Université Laval, Québec City, Canada; 4 Félix d’Hérelle Reference Center for Bacterial Viruses, Faculté de médecine dentaire, Université Laval, Québec City, Canada; 5 Applied Mathematics, Western University, London, Ontario, Canada; University of Georgia, United States of America

## Abstract

The emergence and re-emergence of pathogens remains a major public health concern. Unfortunately, when and where pathogens will (re-)emerge is notoriously difficult to predict, as the erratic nature of those events is reinforced by the stochastic nature of pathogen evolution during the early phase of an epidemic. For instance, mutations allowing pathogens to escape host resistance may boost pathogen spread and promote emergence. Yet, the ecological factors that govern such evolutionary emergence remain elusive because of the lack of ecological realism of current theoretical frameworks and the difficulty of experimentally testing their predictions. Here, we develop a theoretical model to explore the effects of the heterogeneity of the host population on the probability of pathogen emergence, with or without pathogen evolution. We show that evolutionary emergence and the spread of escape mutations in the pathogen population is more likely to occur when the host population contains an intermediate proportion of resistant hosts. We also show that the probability of pathogen emergence rapidly declines with the diversity of resistance in the host population. Experimental tests using lytic bacteriophages infecting their bacterial hosts containing Clustered Regularly Interspaced Short Palindromic Repeat and CRISPR-associated (CRISPR-Cas) immune defenses confirm these theoretical predictions. These results suggest effective strategies for cross-species spillover and for the management of emerging infectious diseases.

## Author summary

The probability that an epidemic will break out is highly dependent on the ability of the pathogen to acquire new adaptive mutations and to induce evolutionary emergence. Forecasting pathogen emergence thus requires a good understanding of the interplay between the epidemiology and evolution taking place at the onset of an outbreak. Here, we provide a comprehensive theoretical framework to analyze the impact of host population heterogeneity on the probability of pathogen evolutionary emergence. We use this model to predict the impact of the fraction of susceptible hosts, the inoculum size of the pathogen, and the diversity of host resistance on pathogen emergence. Our experiments using lytic bacteriophages and CRISPR-resistant bacteria support our theoretical predictions and demonstrate that manipulating the diversity of resistance alleles in a host population may be an effective way to limit the emergence of new pathogens.

## Introduction

Understanding the factors that govern the ability of pathogens to invade a new host population is of paramount importance to design better surveillance systems and control policies. Mathematical epidemiology can provide key insights into these dynamics [1–4]. For instance, simple deterministic models identified critical vaccination thresholds, above which pathogens are driven extinct, which informed policy guidelines for vaccination campaigns [1]. However, chance events and rapid pathogen evolution can also play a critical role in determining the outcome of disease dynamics [2,4–6]. For example, recent experimental studies indicated that the dramatic size of the 2013–2016 Ebola epidemic can at least be partially explained by the acquisition of genetic mutations that increased transmissibility to humans [7,8].

Stochastic models of epidemiology can help to understand the emergence of evolving pathogen populations [5,9–13]. These models, however, often make the unrealistic assumption that the pathogen is spreading in a well-mixed and homogeneous host population, in which all hosts are equally susceptible. Although a handful of theoretical studies have shown that host heterogeneity could have an important impact on pathogen emergence, these models either relied on phenomenological or numerical approaches [12,13], or assumed that the hosts only differ in their number of contacts but not their susceptibility to pathogens [11]. Here, we extend this line of inquiry by (i) building a mechanistic model of pathogen emergence in a diverse host population, in which only some hosts are resistant to the pathogen, (ii) deriving analytical expressions for the probability of evolutionary emergence of the pathogen, and (iii) providing the first experimental test of theoretical predictions on pathogen evolutionary emergence using a bacteria–phage interaction. We demonstrate that realistic increases in the diversity of host resistance alleles strongly reduce the probability of evolutionary emergence of novel pathogens, hence suggesting new strategies to manage the emergence of diseases. Crucially, using bacteria with distinct Clustered Regularly Interspaced Short Palindromic Repeat (CRISPR) immunity and their lytic viruses (bacteriophages) [14–17], we experimentally explore the effect of host population heterogeneity on the emergence and evolution of pathogens. The experimental validation of our theoretical predictions with this microbial system confirms the ability of our mathematical model to capture the complexity of the interplay between the epidemiology and evolution of emerging pathogens in this model system.

## Results

### Theory: Understanding the probability of pathogen emergence

In order to predict how the composition of host populations impacts the probability of pathogen emergence, we developed a branching process model [5,9–13]. We aimed to capture host–pathogen interactions in which different groups of individuals within a host population each carry unique resistance alleles that recognize different pathogen epitopes, and pathogens can evade recognition by acquiring “escape” mutations in the corresponding epitopes. In this model, we assume that the host population contains a fraction (1 − *f*_*R*_) of individuals that are fully susceptible to the pathogen, while the remaining fraction *f*_*R*_ of the population is resistant and composed of a mixture of *n* host types in equal frequencies, each of which has a different resistance allele. The efficacy of resistance is assumed to be perfect (we relax this assumption in section S1.2 of S1 Text). Therefore, a pathogen with *i* escape mutations (*i* between 0 and *n*) can infect a fraction (1 − *f*_*R*_) + *f*_*R*_*i*/*n* of the total host population.

We further assume that a host infected with a pathogen that does not carry escape mutations transmits at rate *b* and dies at rate *d*. Host resistance prevents infection without affecting *b* or *d*. Whereas escape mutations allow the pathogen to infect a larger fraction of the host population, they also carry a fitness cost, *c* which causes pathogens with *i* escape mutations to reproduce at rate *b*_*i*_ = *b*(1 − *c*)^*i*^. The probability of acquiring an escape mutation is a function of *n*, the number of resistance alleles in the population, as well as *i*, the number of escape mutations already encoded by the pathogen. The probability that a pathogen with *i* escape mutations will acquire an additional one equals *u*_*i*,*n*_ = 1 − (1 − *μ*)^*n*−*i*^, where *μ* is the pathogen mutation rate per target site (a target site is a region of the pathogen genome where a point mutation or a deletion may allow escape from recognition by host immunity). This simplifies to *u*_*i*,*n*_ ≈ *μ*(*n* − *i*) when the pathogen mutation rate is assumed to be small (note how the rate of escape mutations increases with (*n* − *i*)). For the sake of simplicity, we assume that escape mutations cannot revert to the ancestral types. These reversions are expected to have a negligible effect on the probability of evolutionary emergence when the target site mutation rate remains small [11]. To account for the effect of spatial structure, we assume that when a pathogen is released from an infected host, it will land with probability *ϕ* on the same type of host (i.e., a host susceptible to this pathogen) and with probability (1 − *ϕ*) on a random host from the population, which may or not be of the same type.

The expected number of secondary infections caused by a pathogen with *i* escape mutations in an uninfected host population is given by its basic reproduction ratio:

$$R_{i,n}=\frac{b_{i}}{d}F_{i,n}$$ (1)

where *F*_*i*,*n*_ = (*ϕ* + (1 − *ϕ*)(*f*_*R*_ *i*/*n* + (1 − *f*_*R*_))) is the effective fraction of hosts that can be infected by the focal pathogen. A pathogen with *n* escape mutations has a basic reproduction ratio equal to *R*_0_(1 − *c*)^*n*^, where *R*_0_ = *b*/*d* refers to the basic reproduction ratio of the pathogen with 0 escape mutations in a fully susceptible host population. Note, however, that a pathogen with 0 escape mutations introduced in a diverse host population has a basic reproduction ratio equal to *R*_0,*n*_ ≤ *R*_0_.

The key question we wish to address with this model is how the composition and structure of the host population determines the ultimate fate of a pathogen (i.e., extinction versus emergence, see S1 and S2 Figs). We detailed in the Materials and methods section the calculation of the probability of emergence, *P*_*i*,*n*_, which is the probability that an inoculum of *V*_0_ pathogens with *i* escape mutations will not go extinct when introduced in a host population with *n* different resistance alleles. To understand the role of pathogen evolution in this process, we also derive the probability of evolutionary emergence, which quantifies the importance of escape mutations to pathogen emergence.

#### Evolutionary emergence is maximized for intermediate proportions of resistant hosts

To understand how the composition of the host population determines the probability that a pathogen emerges, we consider the simple scenario in which the population consists exclusively of sensitive hosts and one type of resistant host with the same resistance allele (i.e., *n* = 1). In this scenario, the probability of emergence of a pathogen with no escape mutation is (see Materials and methods)

$$P_{0,1}=1-{\left(f_{R}+(1-f_{R})\frac{C-\sqrt{-4dA+C^{2}}}{2A}\right)}^{V_{0}}$$ (2)

where *A* = *b*(1 − *μ*)(1 − *f*_*R*_(1 − *ϕ*)), *B* = *bμ* and *C* = *A* + *B*(1 − 1/(*R*_0_(1 − *c*))) + *d*.

In the absence of mutation, the probability of pathogen emergence is thus

$$P_{0,1}^{\mu =0}=1-{\left(f_{R}+\frac{1-f_{R}}{R_{0}(1-f_{R}(1-\phi ))}\right)}^{V_{0}}$$ (3)

**Fig 1:**
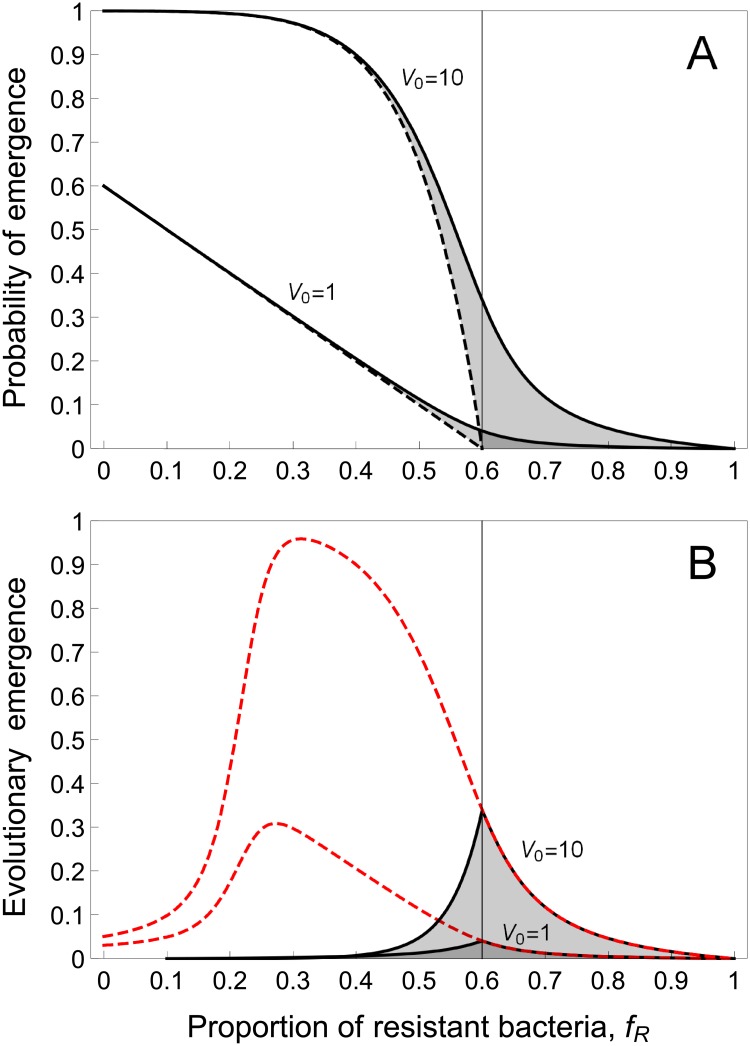
Effect of the proportion of resistant hosts (*f*_*R*_) on pathogen emergence when there is a single type of resistant host (*n* = 1), and for two values of the pathogen inoculum size (*V*_0_ = 1 and 10). (A) Probability of pathogen emergence with (full curve, *u*_0,1_ = 0.01) or without mutations (dashed curve, *u*_0,1_ = 0). The shaded area indicates the effect of pathogen adaptation on emergence. The threshold value *f*_*R*_ = *f*_*T*_ that prevents pathogen emergence in the absence of pathogen adaptation is indicated with a vertical dashed line. (B) Evolutionary emergence of pathogens (the shaded area in A) is maximized for an intermediate value of the fraction of resistant hosts. The dashed red curve shows the theoretical prediction when we account for the change in the escape mutation frequency after the pathogen emergence took place (see section S1.3 of S1 Text). Other parameter values: *b* = 2.5, *d* = 1, *ϕ* = 0, *c* = 0.2, *T* = 24.

Varying the fraction *f*_*R*_ of resistant hosts shows that the probability of emergence decreases linearly with the fraction of resistant hosts in well-mixed populations (when *ϕ* = 0 and *V*_0_ = 1, Fig 1). As expected, the probability of emergence increases with *V*_0_, the size of the pathogen inoculum (Fig 1 and S3 Fig). Population structure has also a positive effect on pathogen emergence, because it helps the pathogen to persist in the susceptible subpopulation. Interestingly, there is a threshold value *f*_*T*_ for the fraction of host resistance, at which the probability of emergence vanishes (Fig 1):

$$f_{T}=\frac{R_{0}-1}{(1-\phi )R_{0}}$$ (4)

Next, we wanted to understand how pathogen evolution can help pathogens emerge. As expected, pathogen mutation generally increases the probability of emergence (unless there is a significant fitness cost associated with escape mutations) because those mutations allow escape from host resistance. The gray area in Fig 1A measures the amount of evolutionary emergence. Interestingly, evolutionary emergence is maximized for intermediate values of the frequency of resistance (usually when *f*_*R*_ = *f*_*T*_, see Fig 1B). Indeed, when the frequency of resistance is low, there is no selection for escape mutations, and those mutations get rapidly lost if they are associated with fitness costs. In contrast, when the frequency of resistance is high, the chains of transmission driven by the wild-type strain are very short and there is a lower rate of appearance of escape mutations. Hence, in spite of the strong selection for escape mutations, the rate of evolutionary emergence is reduced. Intermediate resistance frequency therefore maximizes evolutionary emergence because this is where both the influx and the selection for escape mutations are high. When the host population is spatially structured, the role of pathogen evolution is smaller, simply because the probability of emergence without evolution is higher under these conditions (see S4 Fig).

So far, the model considered only the role of pathogen mutation during the very early stages of the epidemic, when stochastic effects play an important role. However, whenever we observe the emergence of a novel pathogen, these initial stages will usually have already passed, and pathogen evolution may therefore deviate from the patterns that are predicted by the above model. For example, even when escape mutations are not necessary for emergence, those mutations may increase in frequency after the onset of an epidemic. This is particularly true when the proportion of resistant hosts is large, relative to the cost of mutation. After emergence, the size of the pathogen population increases rapidly and one can start to neglect the effect of demographic stochasticity on the change in escape mutation frequencies. To understand how pathogens evolve over longer timescales during an epidemic (i.e., beyond the initial emergence), we combined our stochastic description of pathogen emergence with a deterministic model for the change in the frequency of escape mutations (see section S1.3 of S1 Text). This model predicts that the probability of observing an escape mutation evolving in a pathogen population is maximized for a low frequency of host resistance (red dashed curve in Fig 1B and in S4 and S5 Figs).

#### Diversity of host resistance decreases pathogen emergence

Natural host populations usually consist of multiple host genotypes, each carrying their own resistance allele. To understand how this will impact the predicted patterns of pathogen emergence, we analyzed the situation in which the resistant host population consists of *n* types in equal frequencies, each carrying a unique resistance allele. In the absence of pathogen mutations, emergence is governed by Eq 3 and does not depend on host diversity because all hosts are equally resistant to a pathogen with no escape mutations. Each extra escape mutation, however, allows the pathogen to infect a fraction 1/*n* of the resistant host population, and the pathogen needs *n* escape mutations to exploit the whole host population. Yet, the probability *u*_*i*,*n*_ to acquire an escape mutation increases with *n* because there are more genetic loci (i.e., target sites) involved in the interaction with the host. In other words, both the number of mutations required to reach the top of the fitness landscape and the rate of acquisition of escape mutations increase with the diversity of host resistance. Because these two processes have opposite effects on the rate of pathogen adaptation, it is not immediately obvious how host diversity affects pathogen emergence.

**Fig 2:**
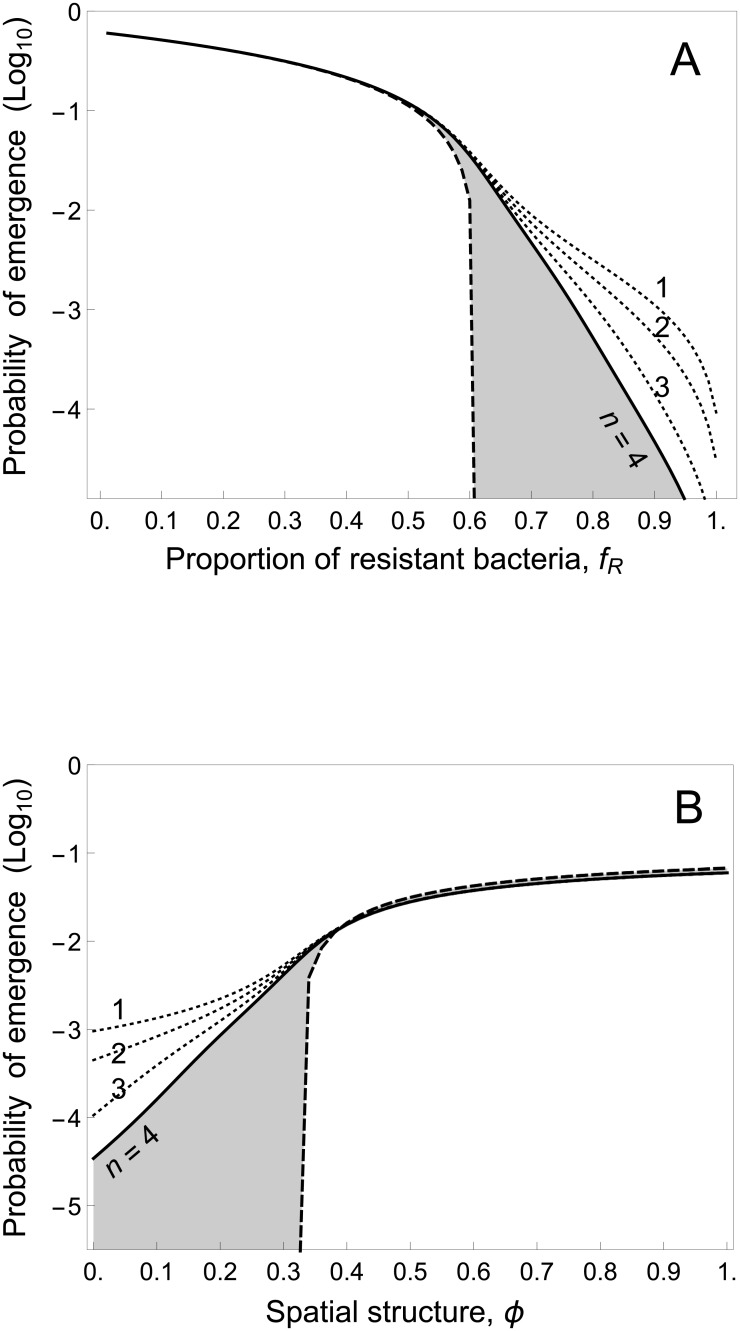
Effect of the fraction of resistant hosts, host resistance diversity (*n*), and spatial structure (*ϕ*) on pathogen emergence. (A) Probability of pathogen emergence without (*u*_0,*n*_ = 0, dashed curve) or with (*u*_0,*n*_ = 5 * 10^−3^, full curve) mutations (note the logarithmic scale) when *n* = 4 and for increasing values of the fraction of resistant hosts in the absence of spatial structure (i.e., *ϕ* = 0). The shaded area illustrates the fraction of pathogen emergence caused by pathogen adaptation. The dotted curves indicate the probability of emergence for lower levels of host diversity. In (B), the probability of pathogen emergence is shown for increasing values of *ϕ* (i.e., spatial structure) when *f*_*R*_ = 0.88. Other parameter values: *b* = 2.5, *d* = 1, *c* = 0.05.

We derive numerically the probability of pathogen emergence under a broad range of scenarios (see Materials and methods). In Fig 2A, we show the joint effects of the frequency of resistance and the diversity of resistance. Increasing host diversity always decreases the probability of emergence of the pathogen. We also find a very strong interaction between host diversity and spatial structure. Because spatial structure induces a clustering of the different types of resistant hosts, it reduces the effective diversity of host resistance and promotes pathogen emergence (Fig 2B).

The above analysis relies on (i) the assumption that the pathogen life cycle can be approximated by the birth–death model and (ii) the assumption that the frequency of host resistance does not vary through time. We relaxed both these assumptions with individual-based simulations in section S1.4 of S1 Text and obtained very similar results (see S6, S7 and S8 Figs).

### Experiments on phage emergence confirm theoretical predictions

Next, we wanted to experimentally explore the validity of the above predictions. While this is challenging given the paucity of suitable empirical systems that are amenable to experimental manipulations in a timely fashion, we explored whether this could be achieved by studying the evolutionary emergence of “escape” phages against bacteria with a CRISPR–CRISPR-associated (Cas) system. This immune defense provides full protection against a phage infection by adding phage-derived sequences (known as “spacers”) in a CRISPR locus carried by the bacterial host chromosome [14]. This empirical system allowed us to overcome three important technical challenges (see details of the experimental protocols in the Materials and methods section). First, the stochastic nature of extinction requires a large number of replicate populations to measure a probability of emergence, which is possible using bacteria and phages in 96-well plates. Second, by mixing bacteria with different and unique CRISPR resistance alleles, we could manipulate the fraction of resistant hosts and the diversity in resistance alleles without affecting other traits of the host [18]. Third, unlike most other empirical systems, the mechanism of phage adaptation to CRISPR-based immunity is well known: lytic phages “escape” CRISPR resistance through mutation of their target sequence (the “protospacer”) [13,15,18,19,20].

**Fig 3:**
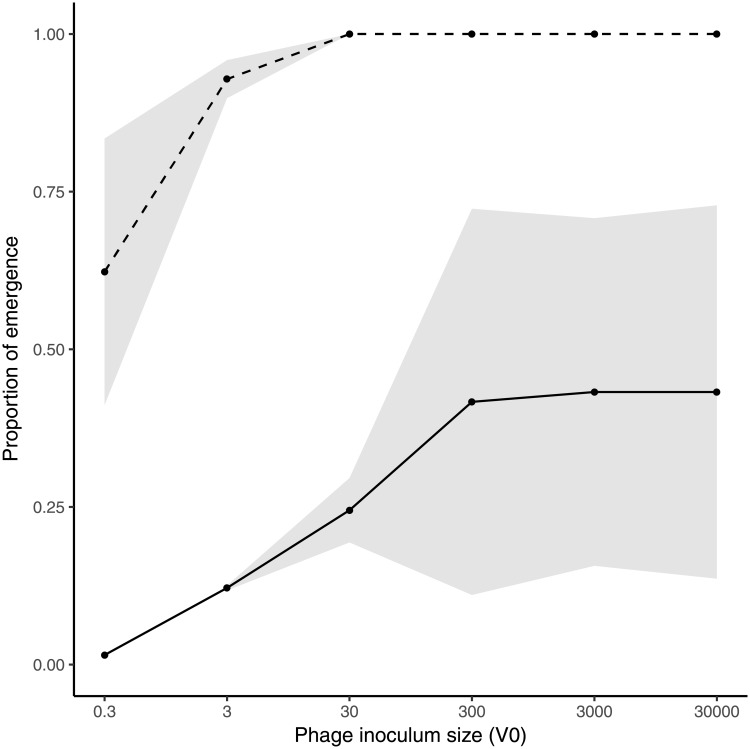
Probability of evolutionary emergence increases with the size of phage inoculum (*V*_0_). The proportion of replicate populations in which emergence (i.e., in which the amplification of the phages is detected, dashed line) or evolutionary emergence (i.e., in which the amplification of an escape phage is detected, solid line) was observed following inoculation with *V*_0_ unevolved phages in 96 independent replicate populations, each consisting of 50% sensitive bacteria and 50% BIMs (*f*_*R*_ = 0.5). The different values of phage inoculum were obtained by serial dilution and correspond approximately equal to *V*_0_ = 0.3, 3, 30, 300, or 3,000, where *V*_0_ refers to the mean of a Poisson distributed number of viruses. Shaded areas represent 95% confidence intervals of the mean of two experiments. Data are available in S1 Data. BIM, bacteriophage-insensitive mutant.

In order to validate the model using this empirical system, we used eight CRISPR-resistant clones (also referred as bacteriophage-insensitive mutants [BIMs]) of the gram-negative *Pseudomonas aeruginosa* strain UCBPP-PA14, each of which carried a single and distinct spacer targeting the lytic phage DMS3vir. Each of these spacers provides full resistance to infection. For each of these eight CRISPR-resistant clones, the rate at which the phage acquires escape mutations was found to be approximately equal to 2.8*10^−7^ mutations/locus/replication, as determined using Luria-Delbrück experiments (see section S2.1.6 in S1 Text and S9 Fig). Using one of these BIMs, we first tested the theoretical prediction that the probability of emergence increases with the size of the virus inoculum (*V*_0_). To this end, 96 replicate populations, each composed of an equal mix of sensitive bacteria and a CRISPR-resistant clone, were exposed with five different inoculum sizes of the phage (corresponding to a mean *V*_*0*_ of approximately 0.3, 3, 30, 300, and 3,000 phages). After 24 hours, we measured the fraction of phage-infected bacterial populations in which emergence had occurred. Consistent with the model predictions, we observed that the larger the phage inoculum size, the higher the probability of pathogen emergence (Fig 3, dashed line). In addition, we measured the fraction of viral populations in which the phages had evolved to escape CRISPR resistance. Again, in accordance with the theory, we found that larger phage inocula were associated with an increased evolution of phage escape mutations (Fig 3, full line, Kendall, z = 3.416, tau = 0.784, *p* < 0.001). Furthermore, we obtained very similar results using a different empirical system consisting of the lytic phage 2972 and its gram-positive bacterial host *Streptococcus thermophilus* DGCC7710. In this experiment, 96 populations composed of sensitive bacteria and a CRISPR-resistant clone were infected with three different inoculum sizes of the phage. As above, we found that a larger phage inoculum led to both a higher probability of emergence and a higher probability of evolutionary emergence (S10 Fig).

**Fig 4:**
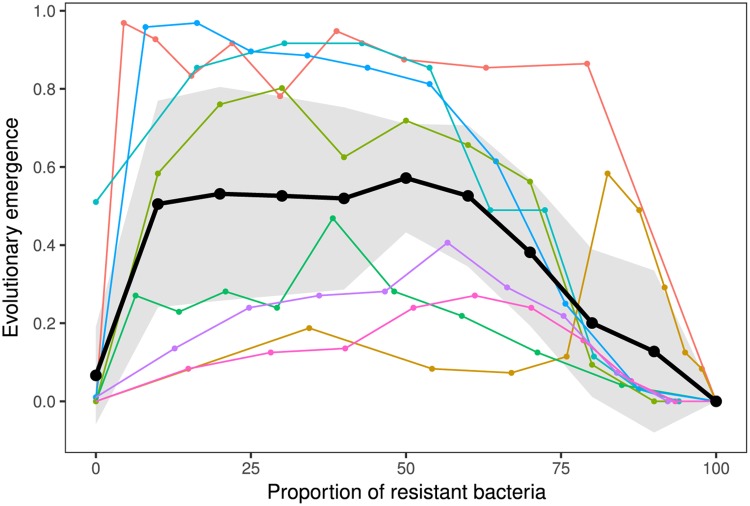
Intermediate proportion of resistant hosts maximizes the probability of evolutionary emergence. Proportion of replicate populations with evolutionary emergence (i.e., in which the amplification of an escape phage is detected) for increasing values of the proportion of resistant bacteria (*f*_*R*_). The different colors correspond to replicate experiments performed using eight different BIMs (see S2 Table and S9 Fig). For each treatment, each of the 96 replicate host populations was inoculated with an initial quantity approximately equal to *V*_0_ = 300 unevolved phages. Black lines indicate the mean across the eight BIMs; gray shaded areas represent 95% confidence intervals of the mean. Data are available in S1 Data. BIM, bacteriophage-insensitive mutant.

Next, we tested the theoretical prediction that the probability of pathogen evolutionary emergence is highest in populations with an intermediate fraction of resistant hosts (Fig 4). For each of the eight BIMs, we generated populations composed of sensitive bacteria and a variable proportion of CRISPR-resistant bacteria, ranging from 0% to 100% in 10% increments. These populations were subsequently infected with *V*_0_ = 300 phages, and the fractions of emergence and evolutionary emergence were measured. As expected, pathogen/phage emergence dropped when the proportion of host/bacteria resistance reached a threshold level (S11 Fig). Interestingly, examination of phage evolution among emerging phage populations also confirmed that the probability of observing escape mutations is maximized for intermediate proportions of host resistance (Fig 4). Again, we obtained very consistent results with phage 2972 and *S*. *thermophilus* (S10 Fig).

We noticed substantial variation among CRISPR-resistant hosts in the observed frequencies of escape phage evolution (Fig 4). Variations in phage mutation rates are unlikely to explain this variability because, as pointed out above, we failed to detect significant variations in the rate of escape mutations to the different CRISPR-resistant hosts (see S9 Fig). Variations in the fitness cost associated with these mutations could, however, explain the observed variations in the final frequency of escape mutations (see S5 Fig).

**Fig 5:**
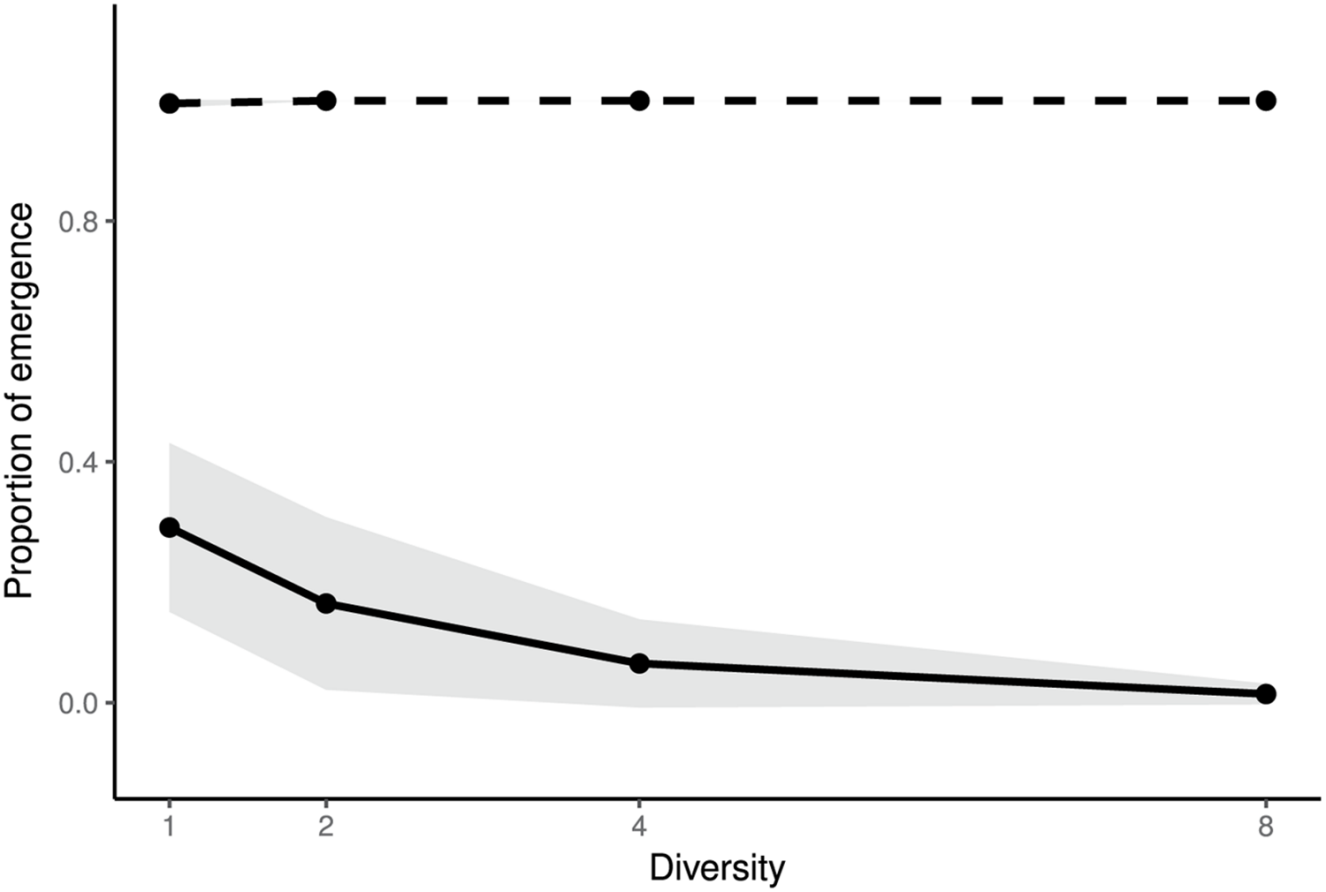
Increasing the diversity of host resistance decreases the probability of evolutionary emergence. Proportion of replicate populations with phage emergence (i.e., in which the amplification of the phages is detected, dashed line) or evolutionary emergence (i.e., in which the amplification of an escape phage is detected, solid line) for increasing values of the diversity of host resistance (*n* = 1, 2, 4, or 8 BIMs) when the proportion of host resistance is *f*_*R*_ = 0.5. For each treatment, each of the 96 replicate host populations was inoculated with an initial quantity approximately equal to *V*_0_ = 300 unevolved phages. Shaded areas represent 95% confidence intervals of the mean of two experiments. Data are available in S1 Data. BIM, bacteriophage-insensitive mutant.

Finally, we experimentally explored the effect of resistance allele diversity on evolutionary emergence for a fixed proportion of host resistance (*f*_*R*_ = 0.5). To this end, we generated bacterial populations that were composed of sensitive bacteria and an equal mix of one, two, four, or eight CRISPR-resistant clones. In this case, as expected, an inoculum size of 300 phages always led to pathogen emergence, but increasing host diversity had a strong negative effect on the ability of the phage to evolve to escape host resistance (Fig 5). We also found higher probabilities of observing multiple escape mutations in the low diversity treatment (Kendall, z = −4.8771, Tau = −0.3259, *p* = 1.07*10^−6^), which further supports the prediction that host diversity hampers the evolution of the phage population.

## Discussion

The emergence and re-emergence of pathogens has far-reaching negative impacts on wildlife, agriculture, and public health. Unfortunately, pathogen emergence events are notoriously difficult to predict and we need good biological models to experimentally explore the interplay between epidemiology and evolution taking place at the early stages of an epidemic. Here, we used a combination of diverse theoretical and experimental analyses to examine how the composition of a host population impacts the probability of pathogen emergence and evolution. Our theory is tailored to the biology of CRISPR–phage interactions, and subsequent validation using this experimental system demonstrates the predictive power of this theoretical framework. However, we suggest that this framework may be suitable for predicting pathogen emergence whenever hosts recognize specific pathogen epitopes and resistance can be overcome by epitope mutations. For instance, the specificity of the host–parasite interaction driven by CRISPR immunity (S12 Fig) is akin to the classical gene-for-gene system described in plant pathosystems [21]. However, host immunity may not always be perfect, which will impact both the dynamics and the evolution of the pathogen population [22–24]. To further generalize our findings, we derived the probability of pathogen emergence when immunity is imperfect (see section S1.2 in S1 Text). Note, however, that this should be considered separately from the more complex epidemiological dynamics that occur when the probability of a successful infection depends on the pathogen dose or when the pathogen causes immunosuppression, both of which can cause emergence to become dependent on the pathogen population density [25,26].

Our framework provides several insights on emergence and re-emergence in both the presence and absence of pathogen evolution. For instance, this model captures how the composition and diversity of the host population impacts the emergence of a nonevolving pathogen. In this context, a larger proportion of resistant hosts decreases pathogen emergence, but this effect is weaker in spatially structured populations in which transmission is more likely to occur between the same host types, which allows for pathogen persistence in sensitive subpopulations. This effect is akin to the effect of the spatial distribution of suitable habitats on extinction thresholds [27–30] and consistent with earlier work that shows that host composition and spatial structure impact the growth rate of bacteriophage ϕ6 [31]. In the context of an evolving pathogen, our theory helps to explain the general observation that evolutionary emergence and the spread of escape mutations is maximal for an intermediate proportion of resistant hosts in the population [32]. Specifically, this is because increasing host resistance in the population has two opposite effects: (i) the influx of new mutations decreases because the ancestral pathogen cannot replicate on resistant hosts, and (ii) selection for escape mutations increases.

Second, our model predicts that diversity in host resistance alleles decreases the probability of evolutionary emergence. Even though larger host diversity increases the number of adaptive mutations for the pathogen (i.e., a larger number of targets of selection), each mutation is associated with a smaller fitness advantage (i.e., a smaller increase in the fraction of the host population that can be infected). The theory presented here therefore helps to explain previous empirical data on the impact of host CRISPR diversity on the evolution of escape phages [18]. The link between host biodiversity and infectious diseases has attracted substantial attention recently [33–43]. Several studies support the “dilution effect” hypothesis, which postulates that host diversity limits disease spread [39,40, 43]. For example, host diversity may limit the spread of a pathogen by increasing the fraction of bad-quality hosts in the population [43]. Indeed, increasing the fraction of resistant hosts (but not the diversity of resistance alleles) decreases the basic reproduction ratio of the wild-type pathogen [44,45]. In addition, host diversity per se may also limit disease spread, and several studies have shown the negative effect of host diversity on the deterministic growth rate of the pathogen under specific patterns of host–parasite specificity [35,46,47].

Notwithstanding these important insights, what sets our theoretical model apart is its ability to understand the factors that impact the initial pathogen emergence, rather than the downstream spread of a pathogen once it has already emerged. Studying this requires stochastic models, which are critical to model the probability of rare events, for example, pathogen spillover across species, including at the human-animal interface [48,49,4,50], the emergence of drug resistance [51,52], the evolution of vaccine resistance [53], and the reversion of live vaccines [54–58]. In all these public health issues, understanding pathogen emergence requires models accounting for the stochastic nature of epidemiological and evolutionary dynamics. The present study focuses on the effect of the diversity of host resistance when each resistant host carries a single resistance allele (i.e., a single spacer in CRISPR). Our joint theoretical and experimental approach could be readily extended to evaluate the impact of the accumulation of multiple resistance alleles in a single host genotype rather than mixing multiple genotypes with a single resistance allele in the host population. The impact of such alternative strategies on the durability of resistance and on disease spread is particularly relevant in agriculture [59,60]. Our work provides a theoretical framework to study these different issues, and our experimental model system can be used to evaluate the ability of different control strategies to limit pathogen adaptation and emergence.

## Materials and methods

### Theory

We detail the derivation of the probability of pathogen emergence presented in the main text (the main parameters of the model are listed in S1 Table). We are interested in the ultimate fate (extinction or not) of a single pathogen with *i* escape mutations dropped into a very large host population with a proportion *f*_*R*_ of resistant hosts. This resistant population is composed of an equal frequency of *n* different resistance genotypes. This free infectious particle first has to infect a host to avoid extinction, and the probability of ultimate extinction of this pathogen is

$$Q_{i,n}=(1-f_{R})q_{i,n}+f_{R}(\frac{i}{n}q_{i,n}+\frac{n-i}{n})$$ (5)

where *q*_*i*,*n*_ is the probability of ultimate extinction of the pathogen when it is currently infecting a host.

Next, we focus on the probability *q*_*i*,*n*_(*t*) at time *t* that a pathogen with *i* mutations in an infected host will ultimately go extinct. In a small interval of time, *dt*, four different events may take place. First, the pathogen may transmit to a new host without additional escape mutations. Second, after a mutation event, the pathogen may transmit a pathogen with *i* + 1 escape mutations to a new host. Third, the infected host (and the pathogen in the host) may die. Fourth, nothing may happen during this interval of time *dt*. Collecting these different terms allows us to write down recursions for the probability *q*_*i*,*n*_(*t*), at time *t*, as a function of the probability *q*_*i*,*n*_(*t* + *dt*) and *q*_*i*+1,*n*_(*t* + *dt*), at time *t* + *dt*:

$$\begin{array}{c} q_{i,n}\left(t\right)=\underset{\begin{array}{c} reproduction\\ without\;mutation \end{array}}{\underset{︸}{A_{i,n}dtq_{i,n}\left(t+dt\right)q_{i,n}\left(t+dt\right)}}+\underset{\begin{array}{c} reproduction\\ with\;mutation \end{array}}{\underset{︸}{B_{i,n}dtq_{i,n}\left(t+dt\right)q_{i+1,n}\left(t+dt\right)}}+\underset{death}{\underset{︸}{d\;dt}}\\ +\underset{no\;event}{\underset{︸}{q_{i,n}\left(t+dt\right)\left(1-A_{i,n}dt-B_{i,n}dt-ddt\right)}} \end{array}$$ (6)

with:

*A*_*i*,*n*_ = *b*_*i*_(1 − *u*_*i*,*n*_)*F*_*i*,*n*_

*B*_*i*,*n*_ = *b*_*i*_*u*_*i*,*n*_*F*_*i*+1,*n*_

*F*_*i*,*n*_ = (*ϕ* + (1 − *ϕ*)(*f*_*R*_ *i*/*n* + (1 − *f*_*R*_))).

The above calculation is based on the assumption that the pathogen never reaches a high prevalence and that the composition of the host population remains constant (i.e., *F*_*i*,*n*_ is assumed to remain constant). In other words, the probabilities *q*_*i*,*n*_(*t*) are assumed to be invariant with time. We can thus set *q*_*i*,*n*_(*t*) = *q*_*i*,*n*_(*t* + *dt*) to obtain a recursion equation that allows us to derive *q*_*i*,*n*_ from *q*_*i*+1,*n*_.

The first term of this recursion gives the probability of extinction, *q*_*n*,*n*_ that a pathogen with *n* escape mutations (a pathogen fully adapted to the novel host population) will go extinct. The heterogeneity of the environment has no impact on a fully adapted pathogen, and its probability of extinction is simply the extinction probability of the birth–death process:

$$q_{n,n}=1/\left(R_{0}{\left(1-c\right)}^{n}\right)$$ (7)

Next, to derive *q*_*n*−1,*n*_ from *q*_*n*,*n*_, we need the recursion equation for *q*_*i*,*n*_. However, we have to distinguish two different scenarios. First, if *A*_*i*,*n*_ = 0, for example, the case of a cell infected by a fully maladapted pathogen (i.e., *i* = 0) in a well-mixed population with no susceptible hosts (i.e., *ϕ* = 0, *f*_*R*_ = 1), we find:

$$q_{i,n}=\frac{d}{d+B_{i,n}(1-q_{i+1,n})}$$ (8)

Second, in the more general scenario, in which *A*_*i*,*n*_ > 0, we have

$$q_{i,n}=\frac{C_{i,n}-\sqrt{-4dA_{i,n}+{C_{i,n}}^{2}}}{2A_{i,n}}$$ (9)

with: *C*_*i*,*n*_ = *A*_*i*,*n*_ + *B*_*i*,*n*_(1 − *q*_*i*+1,*n*_) + *d*.

Knowing *q*_*n*,*n*_ and the above recursion equations, we can derive *q*_*n*−1,*n*_ and next *q*_*n*−2,*n*_… until we get *q*_0,*n*_. We are particularly interested in *q*_0,*n*_ and *Q*_0,*n*_ because these quantities measure the probability of extinction of a pathogen with no escape mutations (in an infected host or as an infectious particle, respectively). Ultimately, we obtain the probability of emergence of an inoculum of *V*_0_ propagules of pathogen with no escape mutations (when *n* = 1, this yields Eq 2 in the main text):

$$P_{0,n}=1-{\left(Q_{0,n}\right)}^{V_{0}}$$ (10)

We show in Fig 2 how the diversity of host resistance affects the probability of pathogen emergence through a reduction of evolutionary emergence. In S4 Fig, we illustrate the interaction between host diversity and spatial structure in pathogen emergence. We show that more spatial structure decreases the impact of host diversity on evolutionary emergence and increases the overall probability of pathogen emergence.

### Experiments

To study the impact of the host population composition on the probability of evolutionary emergence, we used two different microbial systems: (i) the gram-negative *P*. *aeruginosa* and its lytic phage DMS3vir, and (ii) the gram-positive *S*. *thermophilus* and its lytic phage 2972. All the resistant bacteria (i.e., BIMs) derived from the phage-sensitive wild-type strains *P*. *aeruginosa* UCBPP PA14 and *S*. *thermophilus* DGCC7710 rely on CRISPR-Cas immunity for complete resistance against the corresponding phage [14,61].

For all treatments, we performed 96 replicate infections of the corresponding host populations. We manipulated the composition of the host populations by mixing overnight cultures of sensitive bacteria and BIMs in the proportions indicated in the text, figures, and figure legends. Each replicate population was inoculated 1:100 into fresh growth media and infected with a quantity *V*_0_ of phages (the inoculum size), as indicated in the text, figures, and figure legends. After 23 hours, we monitored within each population (i) the occurrence of phage epidemics (i.e., an emergence) and (ii) the presence of escape mutants (i.e., an evolutionary emergence). A detailed description of these experiments is provided in section S2 of S1 Text.

## Supporting information

S1 Text Supplementary information. In this supplementary file, we provide (1) an explicit derivation of the probability of evolutionary emergence and additional theoretical results, and (2) more details on the experimental part of the project.
(PDF)

S1 Fig Summary figure. In this study, we explore the effects of three main components of the composition of the host population on the evolutionary emergence of pathogens.
(TIF)

S2 Fig The three alternative outcomes following the inoculation of the host population by a pathogen. The figure (A) is a schematic representation of the host population before and after the emergence. The dots represent uninfected hosts (pink), hosts infected with the wild-type pathogen (blue), and hosts infected with the escape mutant (red) that can infect resistant hosts (indicated with a black contour line). The figure (B) is a schematic representation of the continuous time branching process that accounts for pathogen transmission to a new host (a vertical line), host recovery/death (a cross), and pathogen mutation (a vertical line connecting an infection by a wild type to an infection by an escape mutant).
(TIF)

S3 FigEffect of the pathogen inoculum size (*V*_0_) on pathogen emergence when there is a single type of resistant host (*n* = 1) for different values of the fraction of resistant hosts (*f*_*R*_).In this figure, we plot *P*_0,1_ against *V*_0_ (dashed lines) and $\sum_{V_{0}=0}^{\infty }\frac{e^{-E[V_{0}]}{E[V_{0}]}^{V_{0}}}{V_{0}!}P_{0,1}$ against *E*[*V*_0_] (full line) under the assumption that the number of phages inoculated follows a Poisson distribution with mean *E*[*V*_0_]. Other parameter values: *b* = 2.5, *d* = 1, *u*_0,1_ = 0.01, *c* = 0.01.(TIF)

S4 Fig Effect of the proportion of resistant hosts (*f*_*R*_) on pathogen emergence when there is a single type of resistant host (*n* = 1) and for two values of the phage inoculum size (*V*_0_ = 1 and 10) when *ϕ* = 0.3. (A) Probability of pathogen emergence without (*u*_0,1_ = 0, dashed curve) or with (*u*_0,1_ = 0.01, full curve) mutations. The shaded area refers to the fraction of pathogen emergence caused by pathogen adaptation. The threshold value *f*_*T*_ of the fraction of resistant hosts preventing pathogen emergence in the absence of pathogen adaptation is indicated with a vertical dashed line. (B) Evolutionary emergence of pathogens (the shaded area in A) is maximized for an intermediate value of the fraction of resistant hosts. The dashed red curve represents the theoretical prediction when we track the change in the frequency of escape mutations after emergence (see section S1.3 in S1 Text). Other parameter values (same as in Fig 1, except for *ϕ*): *b* = 2.5, *d* = 1, *ρ* = 1, *c* = 0.2, *T* = 24.
(TIF)

S5 Fig Effect of the cost of escape mutations (*c*) on evolutionary emergence. The shaded area refers to the fraction of pathogen emergence caused by pathogen adaptation (when *c* = 0.01) and is maximized for an intermediate value of the fraction of resistant hosts. The dashed curves represent the theoretical prediction when we track the change of the frequency of escape mutations after pathogen emergence (see section 1.3 in S1 Text) for three different values of the cost of escape mutations: *c* = 0.01 (red), *c* = 0.2 (orange), and *c* = 0.4 (green). Other parameter values: *b* = 2.5, *d* = 1, *u*_0,*n*_ = 10^−3^, *ϕ* = 0, *ρ* = 1, *T* = 20.
(TIF)

S6 FigResults from individual-based simulations (burst-death life cycle with fixed proportions of resistant hosts): Probability of emergence versus the proportion of resistant bacteria when a single propagule (*V*_0_ = 1) is introduced in a well-mixed population (*ϕ* = 0) with a single type of resistance (*n* = 1).Results are shown in the absence (*u*_0,1_ = 0, crosses) or presence (*u*_0,1_ = 0.1, circles) of mutation for burst sizes of 2, 10, and 50. Other parameter values: $\hat{b}=2.5$, *d* = 1, *c* = 0. Results are shown for 10,000 simulation runs. The solid black line in panel A illustrates results for the birth–death process, demonstrating that the two processes are similar but not precisely equivalent when *B* = 2.(TIF)

S7 FigResults from individual-based simulations (burst-death life cycle with fixed proportions of resistant hosts): Probability of emergence versus the proportion of resistant bacteria when a single propagule (*V*_0_ = 1) is introduced in a well-mixed population (*ϕ* = 0).Results are shown on a log scale in the absence of mutation (*u*_0,1_ = 0, crosses, dotted line) or in the presence of mutation (*u*_0,1_ = 0.005), with one to four types of resistance (*n* = 1, 2, 3, dashed lines; *n* = 4, circles, solid line) for burst sizes *B* = 2, 10, and 20. Other parameter values: $\hat{b}=2.5$, *d* = 1, *c* = 0.05. Results are shown for 100,000 simulation runs.(TIF)

S8 Fig Results from individual-based simulations (birth–death life cycle with evolving proportions of resistant hosts). Upper panels (A and B): The probability of emergence versus the initial proportion of resistant bacteria in a well-mixed population (*ϕ* = 0) with a single type of resistance (*n* = 1). Results are shown in the absence (*u*_0,1_ = 0, crosses) or presence (*u*_0,1_ = 0.1, circles) of mutation, for initial inoculum sizes *V*_0_ = 1 (A) or *V*_0_ = 10 (B). For comparison, the predictions of the branching process approximation are shown (red lines). Lower panels (C and D): the fraction of resistant bacteria among uninfected bacteria at the end of the simulation for *V*_0_ = 1 (C) or *V*_0_ = 10 (D). Note the deviation from the *y* = *x* (dotted) line showing that the frequency of resistance is not constant. Other parameter values: *b* = 2.5, *d* = 1, *c* = 0.2. Results are shown for 100 simulation runs. Each simulation was stopped either when the parasite went extinct or when the maximal simulation time was reached (*t* = 200), depending on which event happened first.
(TIF)

S9 Fig Proportion (1 − *P*_0_) of the 96 replicates in which phage escape mutants were generated in the absence of selection (Luria-Delbrück experiment) after plating the phage against 40 different BIM of *Pseudomonas aeruginosa*. In Figs 4 and 5, we used eight different BIMs (indicated in the gray rectangle, see S2 Table), on which our estimation of the phage mutation rate was based (see section S2.1.6 of S1 Text). Data are available in S1 Data. BIM, bacteriophage-insensitive mutant.
(TIF)

S10 Fig Increased phage inoculum size (*V*_0_) and intermediate proportions of resistant hosts (*f*_*R*_) maximize the probability of evolutionary emergence in the *Streptococcus thermophilus* DGCC 7710–phage 2972 system. The proportion of populations (96 total) infected with either *V*_0_ ≈ 2 (red), *V*_0_ ≈ 20 (green), or *V*_0_ ≈ 200 (blue) phages, in which emergence (A, solid lines) or evolutionary emergence (B, dashed lines) was observed. Shaded areas represent estimated 95% confidence intervals. Data are available in S1 Data.
(TIF)

S11 Fig High proportion of resistant hosts minimizes the probability of emergence in the *Pseudomonas aeruginosa* UCBPP-PA14–phage DMS3vir system. Probability of emergence (i.e., when the amplification of the phage is detected) for increasing values of the proportion of a single resistant bacterium (*f*_*R*_). In contrast, Fig 4 presents the probability of evolutionary emergence (i.e., when the amplification of an escape phage is detected). The different colors correspond to replicate experiments performed using eight different BIMs. For each treatment, each of the 96 replicate host populations were inoculated with an initial quantity of *V*_0_ ≈ 300 unevolved phages. Black lines indicate the mean across the eight BIMs; gray shaded areas represent 95% confidence intervals of the mean. Data are available in S1 Data. BIM, bacteriophage-insensitive mutant.
(TIF)

S12 Fig Fitness landscape of the phage in a bacterial population with *n* = 4 different types of resistance. The figure (A) presents the network of 16 different strains with an increasing number of escape mutations. Arrows indicate possible mutation steps. The figure (B) presents the infectivity of five of these 16 different strains against each of the five different bacterial types (where a cross indicates bacteria resistance).
(TIF)

S1 Table Definitions of the main parameters of the model. (DOCX)

S2 Table Spacer sequences of the eight BIMs of *Pseudomonas aeruginosa* used in our study (see S9 Fig). BIM, bacteriophage-insensitive mutant.
(DOCX)

S1 Data Raw data of the experiments presented in Figs 3, 4 and 5, S9, S10, and S11 Figs. The file contains five tabs and the data in each tab have been used to generate one (or two) figure(s). **(1) Tab** Fig 3. The table is composed of four columns: “Phages” indicates the number of phages initially inoculated, “Fraction” indicates the proportion of replicates (between 0 and 1) in which an epidemic occurred, “Type” indicates whether this epidemic is an emergence or an evolutionary emergence, and “Replicate” indicates in which replicated experiments the data were obtained. **(2) Tab** Fig 4 and S11 Fig. The table is composed of four columns: “Fraction sensitive” indicates the proportion of sensitive bacteria added at the beginning of the experiment, “Epidemics (on 96 replicates)” indicates the number of replicates (between 0 and 96) in which an epidemic occurred, “Type of epidemics” indicates whether this is an emergence or an evolutionary emergence, and “BIM” indicates the name of the BIM. When “Type of epidemic” is “Evolutionary emergence,” the data were used to draw Fig 4, and when it is “Emergence,” the data were used to draw S11 Fig. **(3) Tab** Fig 5. The table contains four columns: “Strain” contains the name of the strain tested, “Diversity” contains the level of diversity tested, “Epidemics on strain” contains the number of replicates (between 0 and 96) on which an epidemic occurred, and “Rep” indicates in which replicated experiments the data were obtained. **(4) Tab** S9 Fig. The column named “BIM” indicates the name of the tested strain, and the column named “Mutants” indicates the number of replicates (between 0 and 96) in which a mutant was detected. The eight BIMs used in the following steps of this study are the BIMs 25, 38, 19, 39, 7, 9, 13, and 16. These eight bacterial strains are referred to as BIMs 1–8, respectively, in S1 Text. **(5) Tab** S10 Fig. The table contains four columns. The column called “Fraction Sensitive” indicates the fraction of sensitive bacteria at the beginning of the experiment, the column called “Epidemics” indicates the number of replicates (between 0 and 96) in which an epidemic occurred, the column called “Phages per replicate” indicates the number of phages inoculated at the beginning of the experiment, and the column “Type of epidemics” indicates whether the observed epidemics correspond to an emergence or an evolutionary emergence. BIM, bacteriophage-insensitive mutant.
(XLSX)

## Abbreviations

BIM: bacteriophage-insensitive mutant
Cas: CRISPR-associated genes
CRISPR: Clustered Regularly Interspaced Short Palindromic Repeat
