# Supplementary material for: Evolutionary emergence of infectious diseases in heterogeneous host populations
Source: PLoS Biol. 2018 Sep 24;16(9):e2006738. doi: 10.1371/journal.pbio.2006738 (PMC6171948; doi:10.1371/journal.pbio.2006738)
Supplement: S1 Text — In this supplementary file, we provide (1) an explicit derivation of the probability of evolutionary emergence and additional theoretical results, and (2) more details on the experimental part of the project. (PDF) [file pbio.2006738.s001.pdf]

Supplementary Information

Outline

1. **Theory** 2

1.1. Approximate recursions 2

1.2. Imperfect immunity 3

1.3. Evolutionary epidemiology after emergence 5

1.4. Individual based simulations 7

1.4.1. Burst-death life cycle 7

1.4.2. Model with an evolving host population 9

2. **Experiments** 10

2.1. *Pseudomonas aeruginosa* protocols 10

2.1.1. Bacterial strains and phage 10

2.1.2. Culture conditions, phage detection and titration 10

2.1.3. Impact of the initial amount of phages 10

2.1.4. Impact of the fraction of resistant genotypes 11

2.1.5. Impact of the diversity of resistant genotypes 11

2.1.6.Fluctuation test 12

2.2. Streptococcus thermophilus protocols 13

2.2.1. Bacterial strains and phage 13

2.2.2. Culture conditions, phage detection and titration 14

2.2.3. Probability of evolutionary emergence 14

2.3. Statistical analyses 15

3. **References** 16

1. Theory

1.1. Approximate recursions

To obtain a better understanding of the recursion (6) it is possible to derive analytic approximations under the realistic assumption that the mutation rate is low. In particular, it is possible to derive the probability of emergence $p_{i,n} = 1 - q_{i,n}$ of a strain with i escape mutations as a function of the probability of emergence of a strain with $i+1$ escape mutations, but two distinct approximations can be derived depending on the basic reproduction ratio of strain i. We note that the underlying branching process is not strictly a birth-death process, since the infected host can either give rise to a new strain i individual, a new mutant individual, or die, and these three events happen at three different rates. However, we can approximate this as a birth-death process for the strain i lineage, during which a (rare) mutant "birth" might occur.

If $R_{i,n} < 1$, the strain i lineage will go extinct, and the expected length of the lineage en route to extinction is $1/(R_{i,n} - 1)$ generations. Each generation lasts of on average $1/d$ time units, during which time strain $i+1$ mutants, that are ultimately destined to emerge, are produced at rate $u_i\, b_i\, F_{i+1}\, p_{i+1,n}$. This gives the following approximation for the emergence probability:

$$p_{i,n} = u_{i,n} \frac{R_{i+1,n}}{(1 - R_{i,n})(1-c)} \underbrace{p_{i+1,n}}_{\substack{emergence \\ after \\ mutation}} \tag{S1-a}$$

If $R_{i,n} > 1$, a similar logic yields :

$$p_{i,n} = \underbrace{\frac{R_{i,n} - 1}{R_{i,n}}}_{\substack{emergence \\ without \\ mutation}} + \frac{u_{i,n}}{R_{i,n}} \left(\frac{R_{i+1,n}}{(1-c)(R_{i,n} - 1)} \underbrace{p_{i+1,n}}_{\substack{emergence \\ after \\ mutation}} - 1 \right) \tag{S1-b}$$

1.2. Imperfect immunity

Here we explore the situation where immunity is imperfect (ρ measures the efficacy of immunity) and resistant hosts can be infected with a probability $(1-\rho)$ by pathogens lacking the corresponding escape mutations. In this case the probability of ultimate extinction of an infectious pathogen with i escape mutations, introduced in the host population becomes:

$$Q_{i,n} = (1-f_R)q_{i,n} + f_R\left(\frac{i}{n}q_{i,n} + \frac{n-i}{n}\Big((1-\rho)q'_{i,n} + \rho\Big)\right) \tag{S2}$$

where $q_{i,n}$ and $q'_{i,n}$ are probabilities of ultimate extinction of the pathogen when it is currently infecting a host that is susceptible (i.e. sensistive) or resistant, respectively. Note that the notion of susceptibility/resistance is conditional to the pathogen (i.e. it depends on the escape mutations carried by the pathogen).

Next, we focus on the probability $q_{i,n}(t)$ and $q'_{i,n}(t)$ at time t that a pathogen with i mutations, currently in an infected host, will ultimately go extinct. In a small interval of time dt four different events may take place. First, the pathogen may transmit to a new host without additional escape mutations. Second, after a mutation event, the pathogen may transmit a pathogen with $i+1$ escape mutations to a new host. Third, the infected host (and the pathogen in the host) may die. Fourth, nothing may happen during this interval of time dt. Note that accounting for all these events is greatly facilitated by the assumption that the frequencies of the different host genotypes is fully determined by f_R and n. Collecting all these different terms allows us to write down recursions for the probability $q_{i,n}(t)$, at time t, as a function of the probability $q_{i,n}(t+dt)$ and $q_{i+1,n}(t+dt)$, at time $t+dt$:

$$\begin{aligned} q_{i,n}(t) = &\underbrace{A_{i,n}dt q_{i,n}(t+dt)}_{\substack{reproduction\\ without\ mutation}} + \underbrace{B_{i,n}dt q_{i,n}(t+dt)}_{\substack{reproduction\\ with\ mutation}} \\ &+ \underbrace{ddt}_{death} + \underbrace{q_{i,n}(t+dt)\big(1-\big(A_{i,n}+B_{i,n}+d\big)dt\big)}_{no\ event} \end{aligned} \tag{S3-a}$$

$$q'_{i,n}(t) = \underbrace{A'_{i,n}dtq'_{i,n}(t+dt)}_{\substack{reproduction \\ without\ mutation}} + \underbrace{B'_{i,n}dtq'_{i,n}(t+dt)}_{\substack{reproduction \\ with\ mutation}}$$

$$+ \underbrace{ddt}_{death} + \underbrace{q'_{i,n}(t+dt)\big(1-\big(A'_{i,n}+B'_{i,n}+d\big)dt\big)}_{no\ event} \tag{S3-b}$$

with:

$$A_{i,n} = b_i\big(1-u_{i,n}\big)\left(F_{i,n}^{S\to S}q_{i,n}(t+dt) + F_{i,n}^{S\to R}q'_{i,n}(t+dt)\right)$$

$$B_{i,n} = b_i u_{i,n}\left(F_{i+1,n}^{S\to S}q_{i+1,n}(t+dt) + F_{i+1,n}^{S\to R}q'_{i+1,n}(t+dt)\right)$$

$$A'_{i,n} = b_i\big(1-u_{i,n}\big)\left(F_{i,n}^{R\to S}q_{i,n}(t+dt) + F_{i,n}^{R\to R}q'_{i,n}(t+dt)\right)$$

$$B'_{i,n} = b_i u_{i,n}\left(\left(F_{i+1,n}^{R\to S} + \frac{\phi}{n-i}\right)q_{i+1,n}(t+dt) + \left(F_{i+1,n}^{R\to R} - \frac{(1-\rho)\phi}{n-i}\right)q'_{i+1,n}(t+dt)\right)$$

$$F_{i,n}^{S\to S} = \left(\phi + (1-\phi)\left(f_R\frac{i}{n} + (1-f_R)\right)\right)$$

$$F_{i,n}^{S\to R} = (1-\phi)(1-\rho)f_R\frac{n-i}{n}$$

$$F_{i,n}^{R\to S} = (1-\phi)\left(f_R\frac{i}{n} + (1-f_R)\right)$$

$$F_{i,n}^{R\to R} = (1-\rho)\left(\phi + (1-\phi)f_R\frac{n-i}{n}\right).$$

The terms $F_{i,n}^{X\to Y}$ refer to the effective fraction of hosts of type Y (susceptible or resistant) that can be infected by a pathogen carrying i escape mutations and currently in a host of type X (susceptible or resistant). Following the approach detailed in the **Materials and Methods** section we can derive the probabilities $q_{i,n}$ and $q'_{i,n}$ and, consequently (using S2) the probability $Q_{i,n}$ of the ultimate extinction of a free living pathogen (with i escape mutations) dropped in a heterogeneous host population.

Note that $q_{i,n} = q'_{i,n}$ when $\phi = 0$. Indeed, in a well-mixed environment the probability of pathogen extinction does not depend on the state (susceptible or resistant) of the infected host because we assumed that immunity only affects the susceptibility of the host (the transmission of pathogens infecting resistant hosts is not affected by imperfect immunity). If we focus on the scenario where $i = 0$, $n = 1$ and $\phi = 0$ we obtain (compare with equation (2) in the main text):

$$P_{0,1} = 1 - \left(\rho f_R + (1-\rho f_R)\frac{C - \sqrt{-4dA + C^2}}{2A}\right)^{V_0} \tag{S4}$$

with $A = b\,(1-\mu/N)(1-\rho f_R)$, $B = b\mu/N$ and $C = A + B\big(1 - 1/\big(R_0(1-c)\big)\big) + d$.

In the absence of mutation, the probability of pathogen emergence is thus (compare with equation (3) in the main text):

$$P_{0,1}^{\mu=0} = 1 - \left(\rho f_R + \frac{1-\rho f_R}{R_0(1-\rho f_R)}\right)^{V_0} \tag{S5}$$

As expected, the probability of emergence decreases with ρ, the efficacy of host resistance. The threshold value f_T for the fraction of resistance where the probability of emergence vanishes becomes (compare with equation (4) in the main text):

$$f_T = \frac{R_0 - 1}{\rho R_0} \tag{S6}$$

1.3. Evolutionary epidemiology after emergence

The branching process model detailed above is used to determine the fate (extinction or emergence) of the pathogen inoculum introduced in the host population. When the pathogen escapes extinction, its population size grows and its dynamics are less sensitive to demographic stochasticity. It thus becomes biologically relevant as well as much more

convenient to model pathogen epidemiology and evolution as a deterministic process. We focus below on the scenario where the host population is a mix of susceptible cells and a single type of fully resistant cells (i.e. $n = 1$). As in the branching process model, we model the pathogen life cycle as a birth-death model and track the densities of hosts infected with the wild type or the escape mutant viruses (w and m, respectively). This yields the following dynamical system:

$$\begin{aligned} \dot{w}(t) &= \Big((1-u)b\big(\phi + (1-\phi)(1-f_R)\big) - d\Big)w(t) \\ \dot{m}(t) &= ubw(t) + (b(1-c) - d)m(t) \end{aligned} \tag{S7}$$

We assume that $w(0) = V_0$ (i.e., the inoculum size of free virus particles) and $m(0) = 0$ (i.e., no escape mutant at the onset of the epidemic). Escape viruses can only appear by mutation at rate u. Escape mutations are assumed to carry a fitness cost c (lower replication/transmission rate). The simplicity of the above model allows us to obtain explicit expressions for the densities of the two types of infected hosts. In particular the frequency of the escape mutant in the pathogen population at time T is:

$$p(T) = \frac{u\big(1 - e^{-b\gamma T}\big)}{u(1 - e^{-b\gamma T}) + \gamma e^{-b\gamma T}} \tag{S8}$$

where $\gamma = -c + u\big(1 - f_R(1-\phi)\big) + f_R(1-\phi)$. This expression clearly shows that when f_R is large relative to the cost of mutation (i.e. $f_R > (c-u)/\big((1-\phi)(1-u)\big)$), $\gamma > 0$ and one expects to see a deterministic increase of the frequency of the escape mutant with time T in the pathogen population. Empirically, we are able to record the existence of escape virus in our bacteria-phage experimental system by monitoring the presence of virus plaques on resistant bacteria. The ability of escape mutant to form plaques are the product of two factors: whether the pathogen emerged, and if so, whether the frequency of resistance in the emerged population was sufficiently high for detection. While $P_{0,1}$ as derived previously gives the probability of emergence (of either mutant or wildtype), $p(T)$ gives the expected frequency of resistance, given emergence was successful. In the simplest case, we can take $p(T)$ as a proxy measure for how detectable resistance will be, given emergence. In this

case, the product $p(T)P_{0,1}$ gives the overall probability of observing resistance. In **Fig 1, S4 Fig** and **S5 Fig** we plot $p(T)P_{0,1}$ as a function of the frequency of host resistance to capture the result of such experiments, i.e. when pathogen populations are exposed to a lawn of resistance bacteria. This figure illustrates that the frequency of pathogen populations able to grow on resistance bacteria is maximal at an intermediate frequency of resistance. Evolutionary emergence is maximized for frequencies of resistance lower than f_T (given in (4)), in particular when the cost of escape mutations is low. We also explored numerically other models in which the probability of detection was an increasing, saturating function of $p(T)$, or models that accounted for the epidemiology and the evolution of the host population, and we recovered qualitatively similar results.

1.4. Individual based simulations

In this section, we use individual-based stochastic simulations to explore the consequences of relaxing two key assumptions of our analysis. First, we investigate the consequences of a more realistic life-cycle to model the dynamics of lytic bacteriophages. Indeed, the above analysis assumes a *birth-death* life cycle where birth (i.e. transmission) and death are independent events that occur at constant rates b and d, respectively. This is quite far from the actual life cycle of a lytic bacteriophages where a single burst of B new virions are produced after lysis (i.e. death) of infected bacteria. Second, our branching-process approximation assumes that the frequency of resistant bacteria is constant through time. The epidemic, however, could select for resistance in the host population, which could in turn affect the probability of emergence.

1.4.1 Burst-death life cycle

In the model described in the main text, each infected host transmits the pathogen to new hosts at a constant rate, such that in the absence of mutation, the model yields a birth-death process. In individual-based simulation, we tested the robustness of our results by

implementing a *burst-death process*, developed to describe the life cycle of lytic bacteriophages [1]. In the burst-death model, each infected host has a constant burst rate, $\hat{b}$, and constant death rate, d. When a burst event occurs, however, the host dies and B pathogen propagules are simultaneously released. Each propagule of type *i* then independently infects a new host with probability $F_{i,n}$, which depends on the spatial structure and proportion of resistant hosts as described in the main text.

Mutations in the burst-death life cycle can occur independently (each propagule in the burst has an independent chance of mutation) or at the level of a template genome (if a mutation occurs, every propagule in the burst shares the mutation). We have investigated both cases and the results are qualitatively very similar; for simplicity, the results below illustrate the latter case only.

For comparison between models, we note that a burst size of two ("parent" host dies, two propagules released) yields a similar, but not identical, stochastic process to the birth-death model described in the main text ("parent" host remains alive, one new infected host produced), taking the burst rate $\hat{b} = b$, where b is the transmission rate in the main text. For larger burst sizes, we set the burst rate $\hat{b} = b/(B-1)$ such that the reproduction number of the fully adapted pathogen strain is equivalent in each case.

S6 Fig shows results analogous to **Fig 1** in the main text, using the burst-death life cycle with varying burst sizes. The grey area highlights the region in which evolutionary emergence is predicted to occur. The solid black line in panel A shows results for the birth-death process described in the main text, comparable to the burst-death model with a burst size of two. Overall, we find that the qualitative results across all burst sizes are very similar, and similar to the results in the main text, showing again that evolutionary emergence is maximized at intermediate levels of resistance. We note that to maintain an equivalent reproduction number, the burst rate is reduced as the burst size increases, and this reduces the overall probability of emergence for larger burst sizes.

Similarly, **S7 Fig** shows results analogous to **Fig 2** in the main text. Here, we demonstrate the effect of increasing host diversity, n, on the probability of emergence. The probability of emergence is plotted against the fraction of resistant hosts, without mutation (crosses, dotted line), or with mutation for $n = 4$ (circles, solid line), or n= 3, 2 and 1 (dashed lines). Again, we see that evolutionary emergence (grey shading) is maximized at intermediate levels of resistance, and that increasing host diversity reduces the probability of emergence. These results are consistent across all burst sizes.

1.4.2. Model with an evolving host population

Another assumption we made in our analysis is that the frequency of the resistance types is constant through time. We relaxed this assumption in individual-based simulations where, upon an infected host's death, the resulting empty site is immediately occupied through reproduction, so that the total host population remains constant. Reproduction occurs by selecting a random susceptible parent in the population, from which the offspring inherits the resistance profile. In contrast, in our original model the resistance profile is attached to the site, so that the new offspring inherits the resistance profile of the previous occupant of the site, which ensures that the frequency of resistant hosts remains constant. In **S8 Fig**, we show that allowing the frequency of resistance to vary over the course of the epidemic does not alter our main qualitative results. The branching process approximation is qualitatively robust because what matters for predicting emergence are the extinction events during the initial steps of the epidemiological dynamics. At the onset of the epidemic, the selection for resistance induced by the spread of the pathogen (when an epidemic takes off) is still very limited because the shift in the frequency of resistance (see panels C and D in **S8 Fig**) occurs *after* the emergence.

2. Experiments

2.1 *Pseudomonas aeruginosa* protocols

2.1.1 Bacterial strains and phage

Pseudomonas aeruginosa UCBPP-PA14 was challenged with phage DMS3vir to generate 40 independent CRISPR-resistant BIM, following protocols described in [2]. All CRISPR resistant strains possess a single spacer in the CRISPR 2 locus. Spacer sequences acquired by each of the 8 BIMs used in this study (see **S9 Fig**) are provided in **S2 Table**. *P. aeruginosa* UCBPP-PA14Δ*csy3*::*lacZ* (LacZ) [3] was used as the "sensitive strain" throughout all experiments, as it can be infected by the virulent phage DMS3vir, and has an inactive CRISPR-Cas system due to deletion of an essential *cas* gene [3].

2.1.2. Cultures conditions, phage detection and titration

Bacteria and phages were grown in LB broth and incubated at 37°C with constant aeration (180 rpm). Phages were isolated by adding chloroform (10% v/v) to lysates followed by 5 minutes centrifugation at maximum speed (13000 rpm). Detection of phage DMS3vir was performed on the chloroform-treated samples by directly spotting 1 µL of sample onto a lawn of *P. aeruginosa* UCBPP-PA14Δ*csy3*::*lacZ* cells followed by overnight incubation at 37°C. For more precise phage titers, lysates were serially diluted in M9 medium (6 g/L of $Na_2HPO_4.7H_2O$, 3 g/L KH_2PO_4, 0.5 g/L NaCl, 1 g/L NH_4Cl) followed by plaque assay using 50 µL of diluted phages and 300 µL of *P. aeruginosa* UCBPP-PA14Δ*csy3*::*lacZ* culture and overnight incubation at 37°C.

2.1.3. Impact of the initial amount of phages

Thirty mL of LB were inoculated with 75 µL of a fresh overnight culture of *P. aeruginosa* UCBPP-PA14Δ*csy3*::*lacZ* and 75 µL of a fresh overnight culture of BIM1 (see **S2 Table**), of which a diluted sample was plated on LB-Agar supplemented with 50 µg/mL of X-gal to verify the ratio of sensitive and resistant clones based on the number of blue and white colonies.

Next, phages were added at a final titre of 0.3, 3, 30, 300, 3000 or 30 000 phages/200 µL. This 30 mL master mix was then split into 96 replicates of 200 µL each. Each replicate experiment was incubated for 23 hours at 37°C while shaking (at 180 rpm). After incubation, phage detection on *P. aeruginosa* UCBPP-PA14Δ*csy3*::*lacZ* and BIM1 was performed as described above. Spot assay on the *P. aeruginosa* UCBPP-PA14Δ*csy3*::*lacZ* strain detected epidemics due to both emergence and evolutionary emergence whereas the spot assay on the BIM1 strain detected evolutionary emergence only. The entire experiment (with 96 replicates) was replicated twice.

2.1.4. Impact of the fraction of resistant genotypes

We generated bacterial populations that consisted of a mix of one BIM and *P. aeruginosa* UCBPP-PA14Δ*csy3*::*lacZ* at ratios 0:100, 10:90, 20:80, 30:70, 40:60, 50:50, 60:40, 70:30, 80:20, 90:10 and 100:0. Then, 150 µL of each mixture was used to inoculate 30 mL of LB, which was then infected with phages at a final titer of 300 phages/200 µL. This master mix was then split into 96 replicates of 200 µL each, and each replicate was incubated at 37°C during 23 hours while shaking at 180 rpm. After incubation, phage detection was performed as described above. In addition, the ratio of bacterial strains in the mixture with 50% of resistant hosts was estimated as described above. The experiments were carried out with 8 different CRISPR-resistant strains (BIM1-8, see **S2 Table**). To confirm that the phage mutation rates for each of the different sequences targeted by the different BIMs were identical, fluctuating tests were performed following the Luria-Delbrück protocol [4] (see below).

2.1.5. Impact of the diversity of resistant genotypes

Bacterial mixtures composed of 50% of *P. aeruginosa* UCBPP-PA14Δ*csy3*::*lacZ* and 50% of an equal mix of CRISPR-resistant clones (see **S2 Table**) were generated, with ratios confirmed by plating on X-gal LB-plates as described above. We generated different CRISPR diversity levels by mixing 1, 2, 4 or 8 CRISPR resistant clones. For the CRISPR monoculture

(diversity of 1), we tested each of the 8 different BIMs once. For the treatments containing two CRISPR resistant clones, we generated equal mixes of BIM1-2, BIM3-4, BIM5-6, and BIM7-8. For the treatments containing 4 CRISPR resistant clones, we generated equal mixes of BIM1-4, and BIM5-8. For the treatments containing eight CRISPR resistant clones, we generated an equal mix of BIM1-8. This experimental design made sure that each of the BIM used is represented once in each diversity treatment (hence each BIM contributed equally to the mean of all treatments, avoiding sampling biases). For each mixture, 30 mL of LB was inoculated with 150 µL of the bacterial mix and phages at a final titre of 300 phages/200 µL. This master mix was then split into 96 replicates of 200 µL each followed by incubation at 37°C for 23 hours while shaking at 180 rpm and downstream analysis after chloroform treatment was performed as described above. In addition, all bacterial mixtures were titrated on X-gal LB-Agar plates. The entire experiment was duplicated.

2.1.6. Fluctuation test

The probability of a phage to bypass the resistance found in each of the 8 BIM used in this study (see **S2 Table**) was measured using a Luria-Delbrück protocol [4]. Briefly, we developed a three-step protocol that starts with an amplification step of replicate populations of the ancestral phage (with no detectable escape mutations) in liquid cultures of the sensitive/host bacteria (absence of selection for escape mutations). In a second step, 1 µL of each of these lysates is inoculated into a replicate population of liquid culture of a CRISPR-resistant bacterium (BIM). Finally, after this second incubation, 1 µL of each of these replicate populations is plated on a lawn of the corresponding BIM in order to measure the proportion of those replicate populations where escape mutations emerged during the amplification on the sensitive bacteria (first step).

We first set up 96 independent amplifications of phage DMS3vir by infecting 200 µL of a liquid culture of *P. aeruginosa* UCBPP-PA14Δ*csy3*::*lacZ* in LB with $N_i = 300$ phages. After a 24-hour incubation at 37°C at 180 rpm, the culture was treated with chloroform, as described above. The number of phages obtained after this amplification was quantified from

12 randomly chosen replicates: $N_f = 3.75 * 10^8$ phages. Second, 1 µL of each of these 96 replicates is inoculated into 96 replicate populations of 200 µL of liquid cultures of a BIM (40 different BIMs were assessed). The proportion P_0 of replicates in which escape phages did not emerge was monitored by plating 1 µL of each well on a lawn of the corresponding BIM (see **S9 Fig**).

The rate of escape mutations can be estimated using: $\hat{\mu} = -ln(P_0)/\left(z\left(N_f - N_i\right)\right)$ where z is the fraction of the phage population sampled from the first amplification to initiate the second amplification (in our experiment: $z = 1/200$). Indeed, the proportion of wells with no escape mutations is: $P_0 = \sum_{k=0}^{\infty} Q_k (1-z)^k = e^{-z\lambda}$, where Q_k is the probability that k mutants are present in the total phage population after the amplification [5]. The number of mutants should be approximately equal to $Q_k = P(k|\lambda)$ where P is the Poisson distribution with mean $\lambda = \mu\left(N_f - N_i\right)$, and μ is the mutation rate per target site. Pooling the data obtained from 8 different BIM with similar values of P_0 (see **S9 Fig** and **S2 Table**) we obtained an average mutation rate: $\hat{\mu} = 2.8\ 10^{-7} \pm 4.3\ 10^{-9}$ mutations/locus/replication.

2.2 *Streptococcus thermophilus* protocols

2.2.1. Bacterial strains and phage

We used the virulent phage 2972 [6], infecting the strain *Streptococcus thermophilus* DGCC 7710. Strain *S. thermophilus* DGCC 7710 possesses two active Type II-A CRISPR-Cas systems that can respond to a 2972 infection [7]. A single CRISPR-resistant strain was generated by exposing a lawn of sensitive bacteria to a high amount of phages and the plates incubated for two days at 40°C in poor oxygen environment. After incubation, we picked single colonies and streaked them twice. The CRISPR loci from the two active systems were then amplified as in [8]. The CRISPR1 locus that integrated a new single spacer at the leader-end was sequenced using Sanger sequencing (Eurofins Genomics). The sequence of the integrated spacer is 5'-AGAAGCACCTCTTGCGTTGATAAAAGTATT-3'

and is targeting a protospacer in the *orf37* of phage 2972, a gene coding for a putative phage replication protein.

2.2.2. Cultures conditions, phage detection and titration

Bacteria were grown in LM17 medium supplemented with $CaCl_2$ (37 g/L of M17 Oxoid Medium supplemented with 5 g/L of lactose and 10 mM sterile $CaCl_2$) and incubated at 40°C. To detect the presence of phages, square Petri plates (120 mm x 120 mm) were poured with 60 mL of hard agar (LM17+$CaCl_2$ supplemented with 15 g/L of agar). When dried, 400 µL of bacteria in mid-exponential phase mixed in 12 mL of soft agar (LM17+ $CaCl_2$ supplemented with 8 g/L of agar) were added on top of this lawn. Then, 2 µL of a phage solution was spotted on this second layer and the plates were incubated overnight at 40°C in plastic bags.

Phages were titrated by mixing 1 mL of LM17+$CaCl_2$, 400 µL of sensitive bacteria in mid-exponential phase, with 50 µL of phage diluted in buffer (50 mM Tris-HCl pH 7.5, 100 mM NaCl, 8 mM $MgSO_4$) and 6 mL of Soft Agar. This mixture was poured on round Petri plates previously filled with 30 mL of Hard Agar and plates were incubated overnight at 40°C in plastic bags.

2.2.3. Probability of evolutionary emergence

The sensitive strain *S. thermophilus* DGCC 7710 and the derived CRISPR-resistant strain (BIM) were incubated until early stationary phase. Then, 11 populations composed of different proportions of the sensitive and the resistant strains (from 0 to 100% with 10% steps) were created. Then, 300 µL of each population were added to 30 mL of LM17+$CaCl_2$ supplemented with phages at a final titre of 2, 20 or 200 phages/200 µL. Each mixture was then separated into 96 different 200 µL replicates and incubated for 22 hours as described above.

After incubation, 2 µL of each replicate was spotted on both the sensitive and the resistant strains. When plaques were observed, the well was noted as positive; if not as

negative. The test on the sensitive strain detects both emergence and evolutionary emergence whereas the test on the resistant strain detects only evolutionary emergence. The effective proportion of the resistant bacteria used in **S10 Fig** was determined after independent titration of the both the sensitive and the resistant bacteria used to make the different treatments.

2.3. Statistical Analyses

To demonstrate that the likelihood of bypassing host immunity were not different for the 8 *Pseudomonas* BIMS used for this study, we used a binomial regression to predict the likelihood of successful and unsuccessful infections as a function of the BIM genotype. The impact of phage inoculum size on the probability of evolutionary emergence (V_0) was assessed using a Kendall test. To study the impact of the fraction of resistant genotype in the population, we used a mixed model that explains the fraction of evolutionary emergence as the function of the fraction of resistant host including resistant strain (BIM genotype) as a random effect. Evolutionary emergence was fitted as a quadratic term to best fit the data. To assess the impact of resistant allele diversity, we used a generalized linear mixed model that explains the fraction of evolutionary emergence as a function of a diversity (fixed effect) including BIM genotype as a random effect and using a Poisson error structure. To assess the impact of diversity of resistant strains on the probability of multi-evolutionary emergence, we used a Kendall test. Similarly, for the *S..thermophilus* data, we used a generalized linear model that explains the fraction of evolutionary emergence as a function of the phage inoculum size and the squared proportion of resistant host. All statistical analyses were carried with R Software (version 3.3.3) through RStudio (version 1.0.136) and mixed effect models were constructed using the lme4 package [9]. In all cases model residuals were checked for normality and approximate p-values were calculated using the lmerTest package [10].

3. References:

1. Hubbarde JE, Wild G, Wahl LM. Fixation probabilities when generation times are variable: the burst death model. Genetics. 2007;176: 1703–1712. doi:10.1534/genetics.107.072009

2. Westra ER, van Houte S, Oyesiku-Blakemore S, Makin B, Broniewski JM, Best A, et al. Parasite exposure drives selective evolution of constitutive versus inducible defense. Curr Biol. 2015;25: 1043–1049. doi:10.1016/j.cub.2015.01.065

3. Cady KC, Bondy-Denomy J, Heussler GE, Davidson AR, O'Toole GA. The CRISPR/Cas adaptive immune system of *Pseudomonas aeruginosa* mediates resistance to naturally occurring and engineered phages. J Bacteriol. 2012;194: 5728–5738. doi:10.1128/JB.01184-12

4. Luria SE, Delbrück M. Mutations of bacteria from virus sensitivity to virus resistance. Genetics. 1943;28: 491–511.

5. Bradwell K, Combe M, Domingo-Calap P, Sanjuán R. Correlation between mutation rate and genome size in riboviruses: mutation rate of bacteriophage Qβ. Genetics. 2013;195: 243–251. doi:10.1534/genetics.113.154963

6. Lévesque C, Duplessis M, Labonté J, Labrie S, Fremaux C, Tremblay D, et al. Genomic organization and molecular analysis of virulent bacteriophage 2972 infecting an exopolysaccharide-producing Streptococcus thermophilus strain. Appl Environ Microbiol. 2005;71: 4057–4068. doi:10.1128/AEM.71.7.4057-4068.2005

7. Barrangou R, Fremaux C, Deveau H, Richards M, Boyaval P, Moineau S, et al. CRISPR provides acquired resistance against viruses in prokaryotes. Science. 2007;315: 1709–1712. doi:10.1126/science.1138140

8. Hynes AP, Rousseau GM, Lemay M-L, Horvath P, Romero DA, Fremaux C, et al. An anti-CRISPR from a virulent streptococcal phage inhibits *Streptococcus pyogenes* Cas9. Nat Microbiol. 2017;2: 1374–1380. doi:10.1038/s41564-017-0004-7

9. Bates D, Mächler M, Bolker B, Walker S. Fitting linear mixed-effects models using lme4. Journal of Statistical Software. doi:10.18637/jss.v067.i01

10. Kuznetsova A, Brockhoff PB, Christensen RH. B. lmerTest package: tests in linear mixed effects models. Journal of Statistical Software. doi:10.18637/jss.v082.i13
