# Supplementary figures and images for: Evolutionary emergence of infectious diseases in heterogeneous host populations
Source: PLoS Biol. 2018 Sep 24;16(9):e2006738. doi: 10.1371/journal.pbio.2006738 (PMC6171948; doi:10.1371/journal.pbio.2006738)

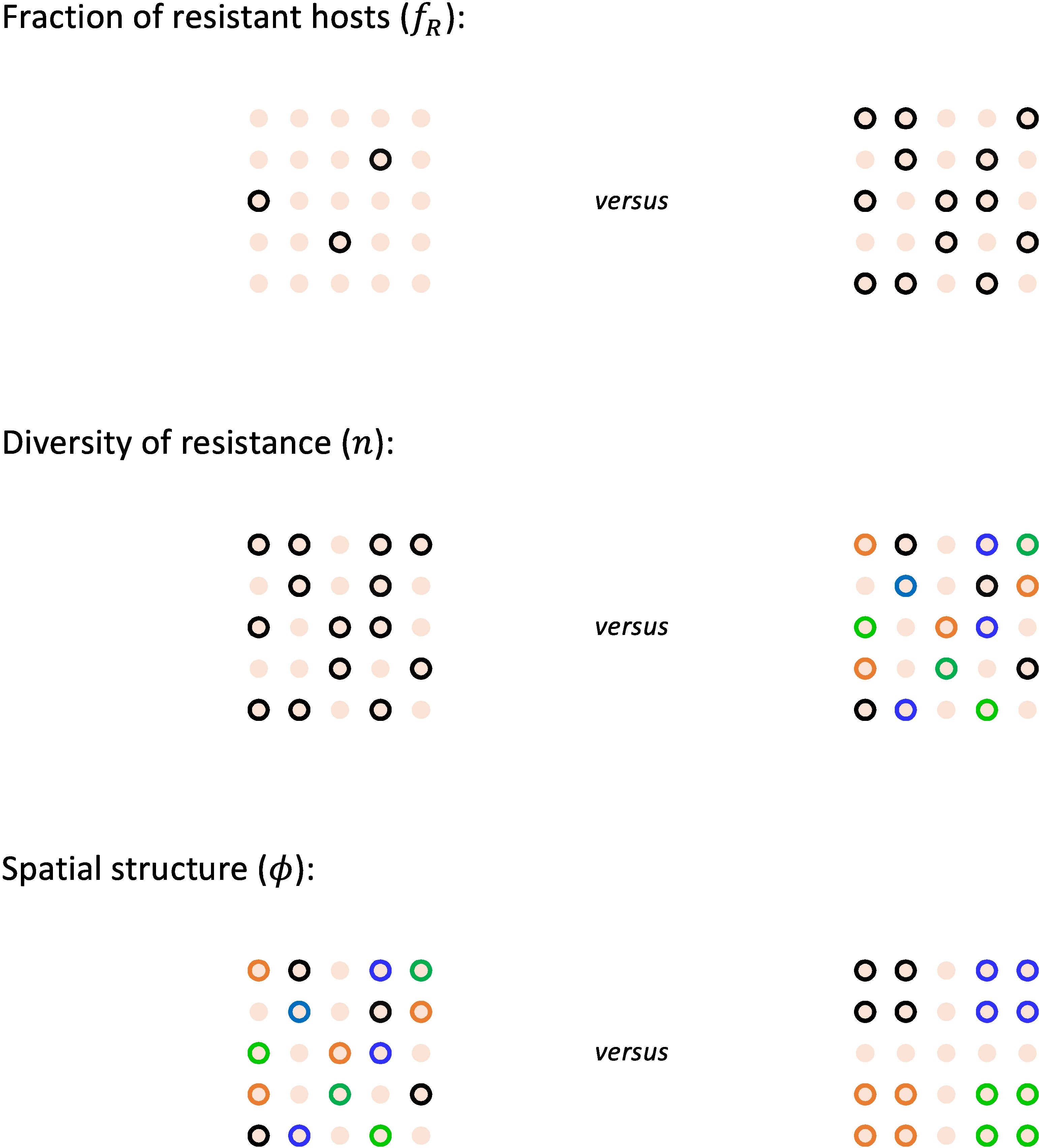

Supplement: S1 Fig — In this study, we explore the effects of three main components of the composition of the host population on the evolutionary emergence of pathogens. (TIF) [file pbio.2006738.s002.tif]

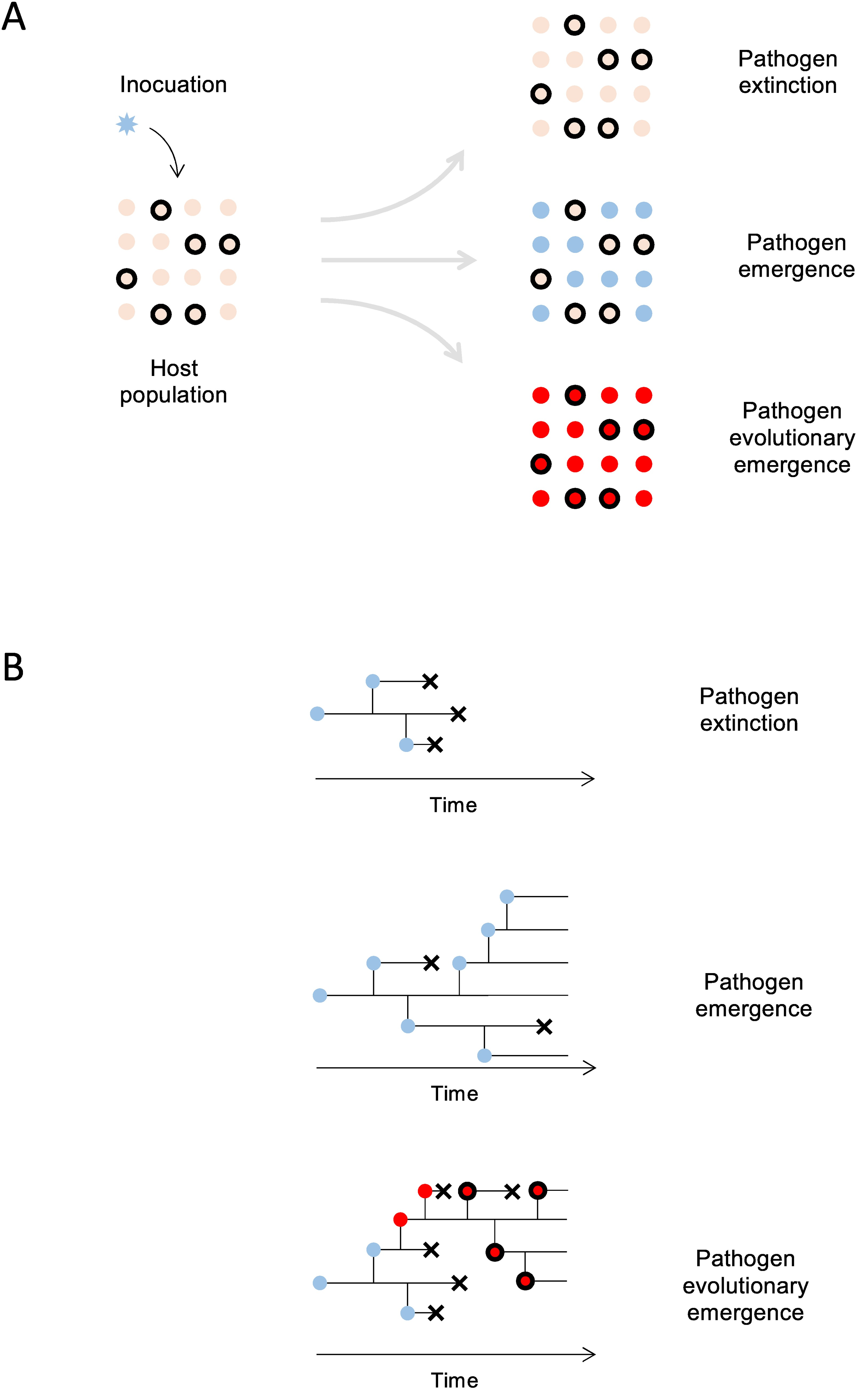

Supplement: S2 Fig — The figure (A) is a schematic representation of the host population before and after the emergence. The dots represent uninfected hosts (pink), hosts infected with the wild-type pathogen (blue), and hosts infected with the escape mutant (red) that can infect resistant hosts (indicated with a black contour line). The figure (B) is a schematic representation of the continuous time branching process that accounts for pathogen transmission to a new host (a vertical line), host recovery/death (a cross), and pathogen mutation (a vertical line connecting an infection by a wild type to an infection by an escape mutant). (TIF) [file pbio.2006738.s003.tif]

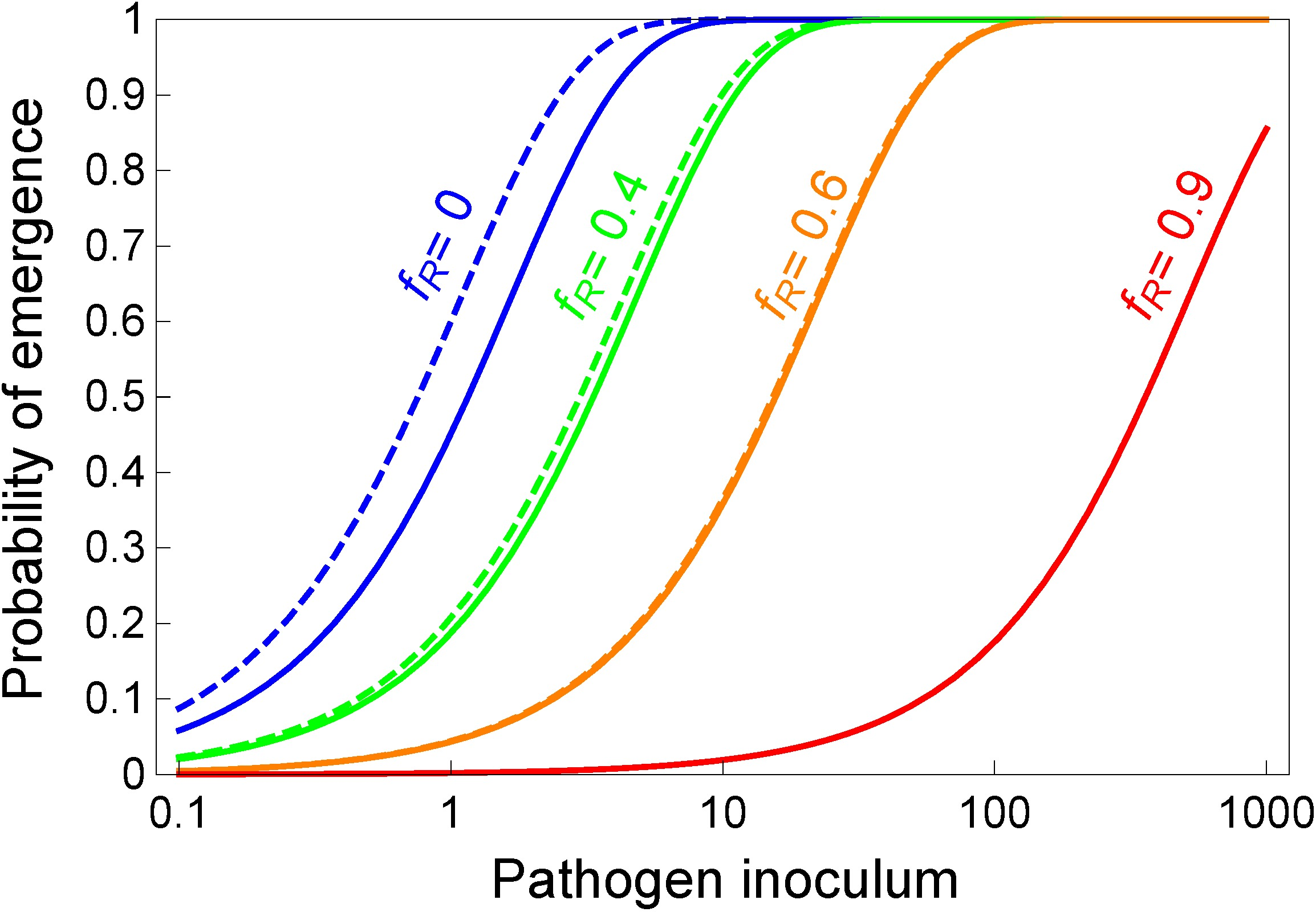

Supplement: S3 Fig — In this figure, we plot P0,1 against V0 (dashed lines) and ∑V0=0∞e-E[V0]E[V0]V0V0!P0,1 against E[V0] (full line) under the assumption that the number of phages inoculated follows a Poisson distribution with mean E[V0]. Other parameter values: b = 2.5, d = 1, u0,1 = 0.01, c = 0.01. (TIF) [file pbio.2006738.s004.tif]

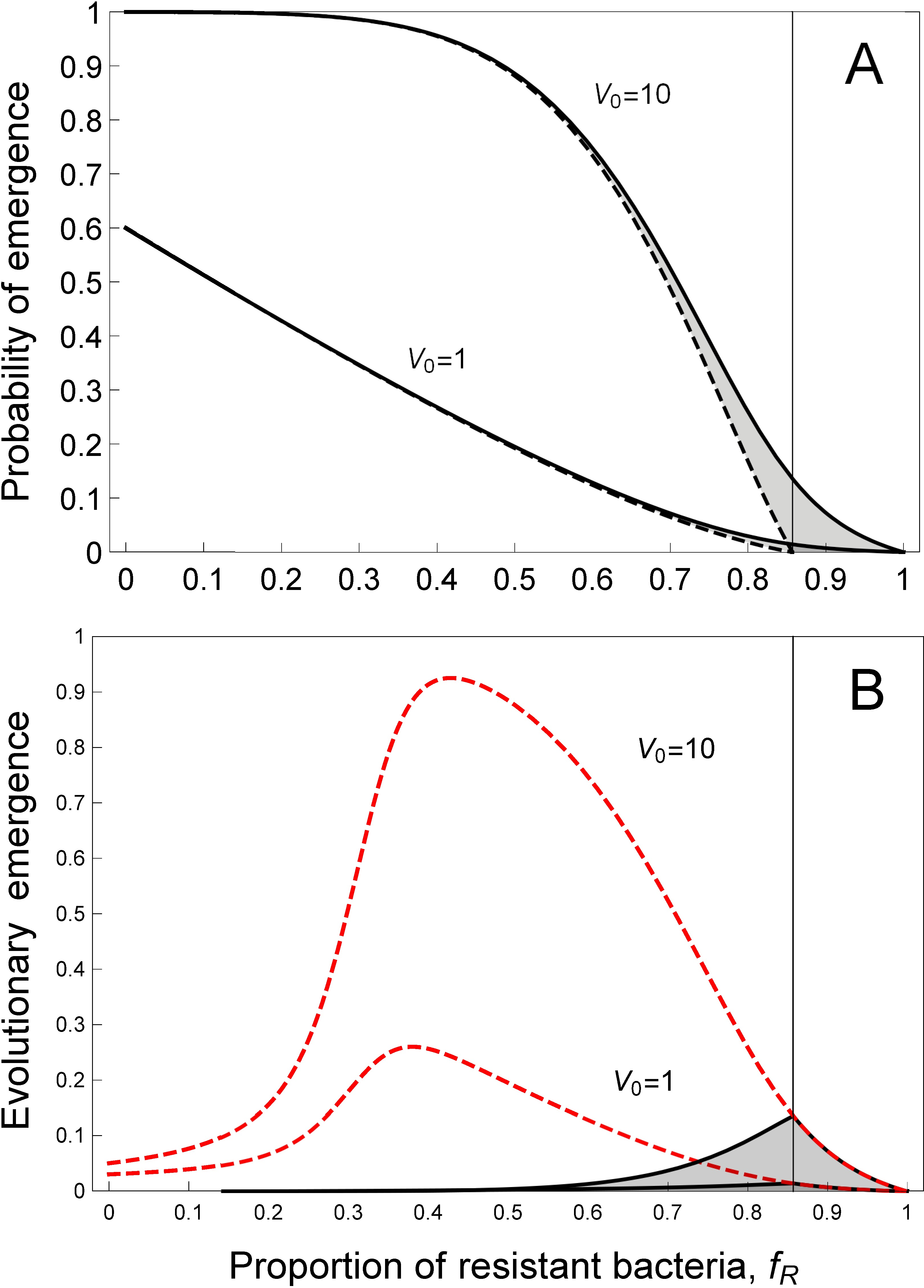

Supplement: S4 Fig — (A) Probability of pathogen emergence without (u0,1 = 0, dashed curve) or with (u0,1 = 0.01, full curve) mutations. The shaded area refers to the fraction of pathogen emergence caused by pathogen adaptation. The threshold value fT of the fraction of resistant hosts preventing pathogen emergence in the absence of pathogen adaptation is indicated with a vertical dashed line. (B) Evolutionary emergence of pathogens (the shaded area in A) is maximized for an intermediate value of the fraction of resistant hosts. The dashed red curve represents the theoretical prediction when we track the change in the frequency of escape mutations after emergence (see section S1.3 in S1 Text). Other parameter values (same as in Fig 1, except for ϕ): b = 2.5, d = 1, ρ = 1, c = 0.2, T = 24. (TIF) [file pbio.2006738.s005.tif]

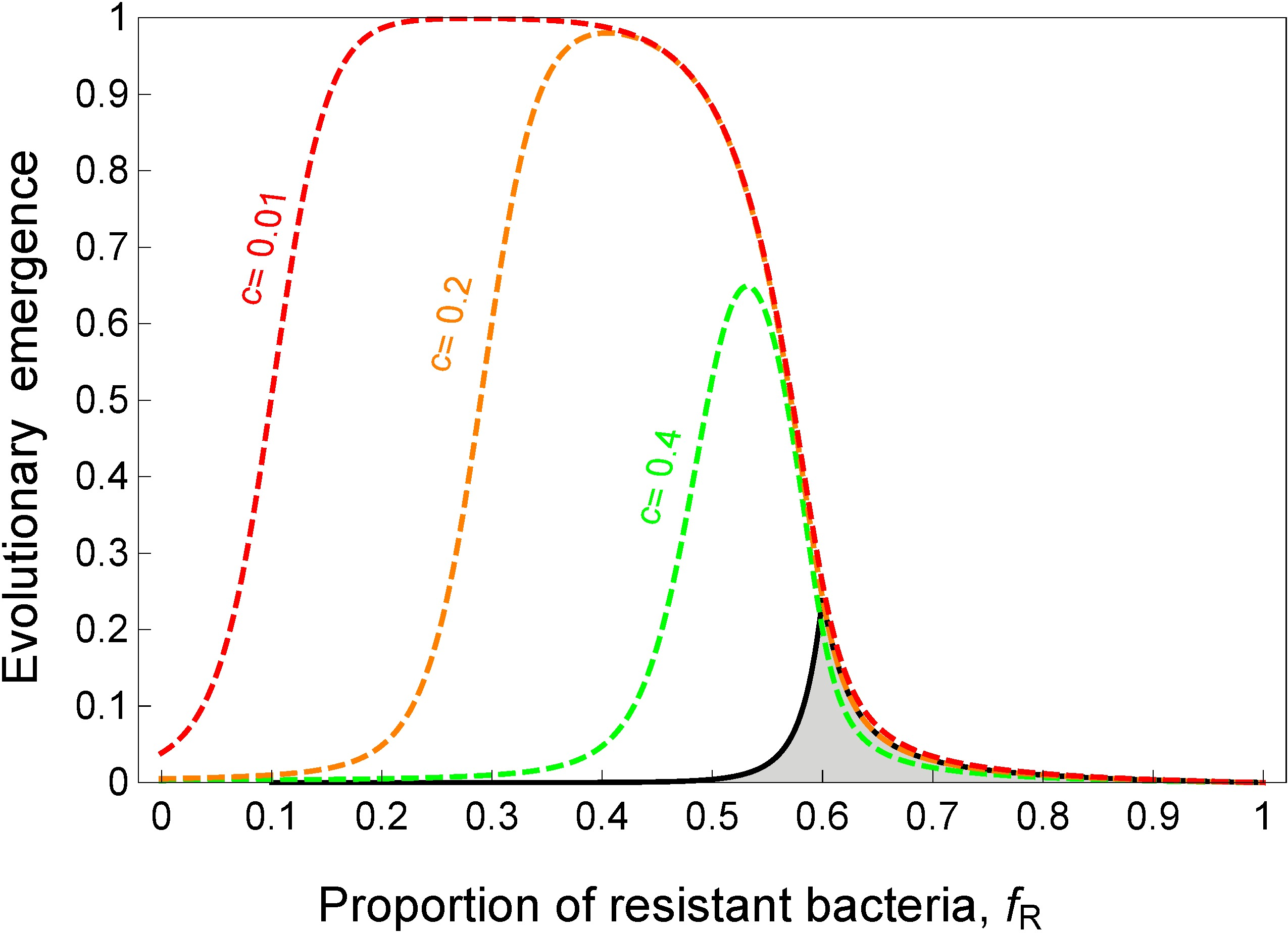

Supplement: S5 Fig — The shaded area refers to the fraction of pathogen emergence caused by pathogen adaptation (when c = 0.01) and is maximized for an intermediate value of the fraction of resistant hosts. The dashed curves represent the theoretical prediction when we track the change of the frequency of escape mutations after pathogen emergence (see section 1.3 in S1 Text) for three different values of the cost of escape mutations: c = 0.01 (red), c = 0.2 (orange), and c = 0.4 (green). Other parameter values: b = 2.5, d = 1, u0,n = 10−3, ϕ = 0, ρ = 1, T = 20. (TIF) [file pbio.2006738.s006.tif]

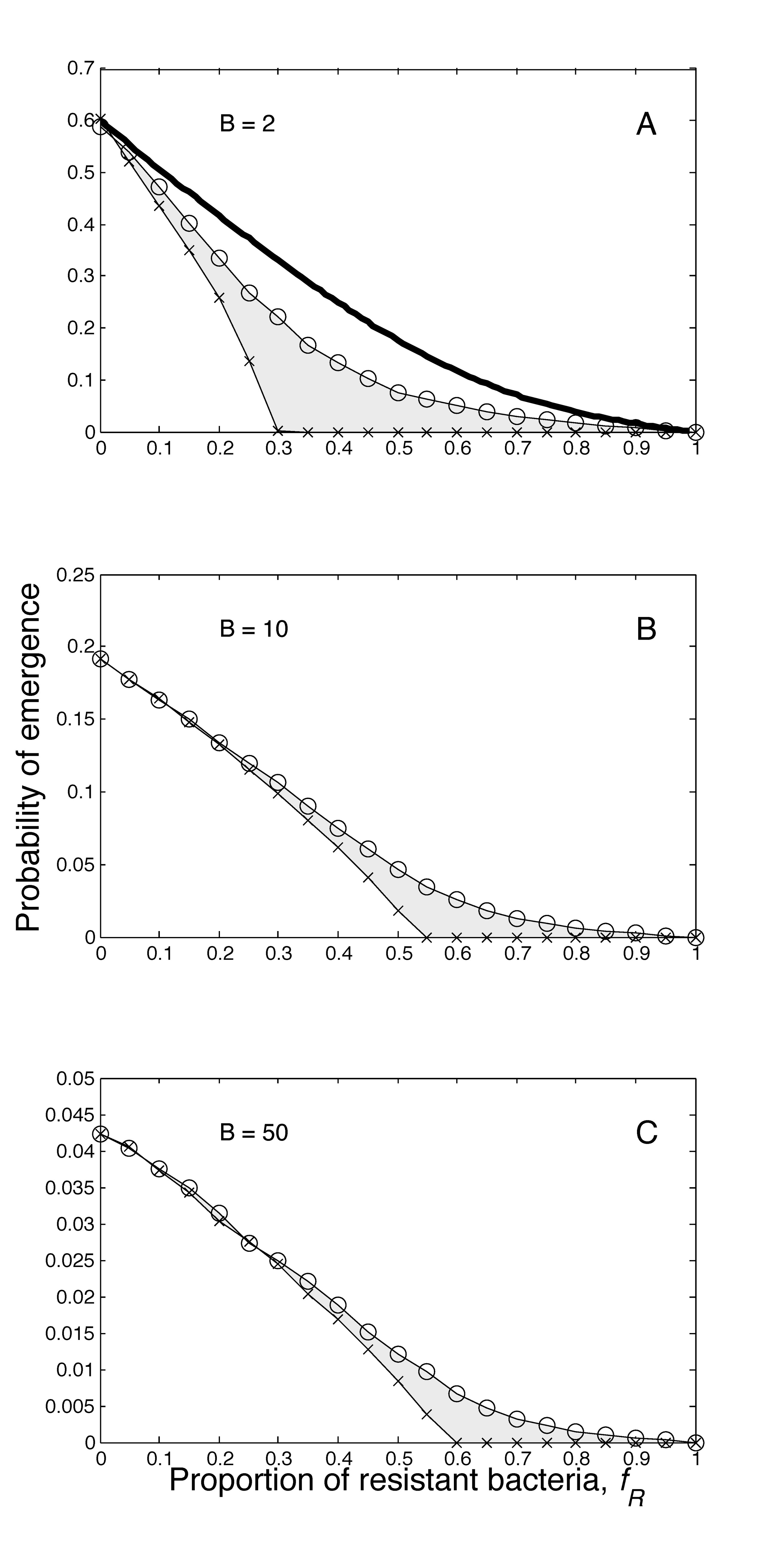

Supplement: S6 Fig — Results are shown in the absence (u0,1 = 0, crosses) or presence (u0,1 = 0.1, circles) of mutation for burst sizes of 2, 10, and 50. Other parameter values: b^=2.5, d = 1, c = 0. Results are shown for 10,000 simulation runs. The solid black line in panel A illustrates results for the birth–death process, demonstrating that the two processes are similar but not precisely equivalent when B = 2. (TIF) [file pbio.2006738.s007.tif]

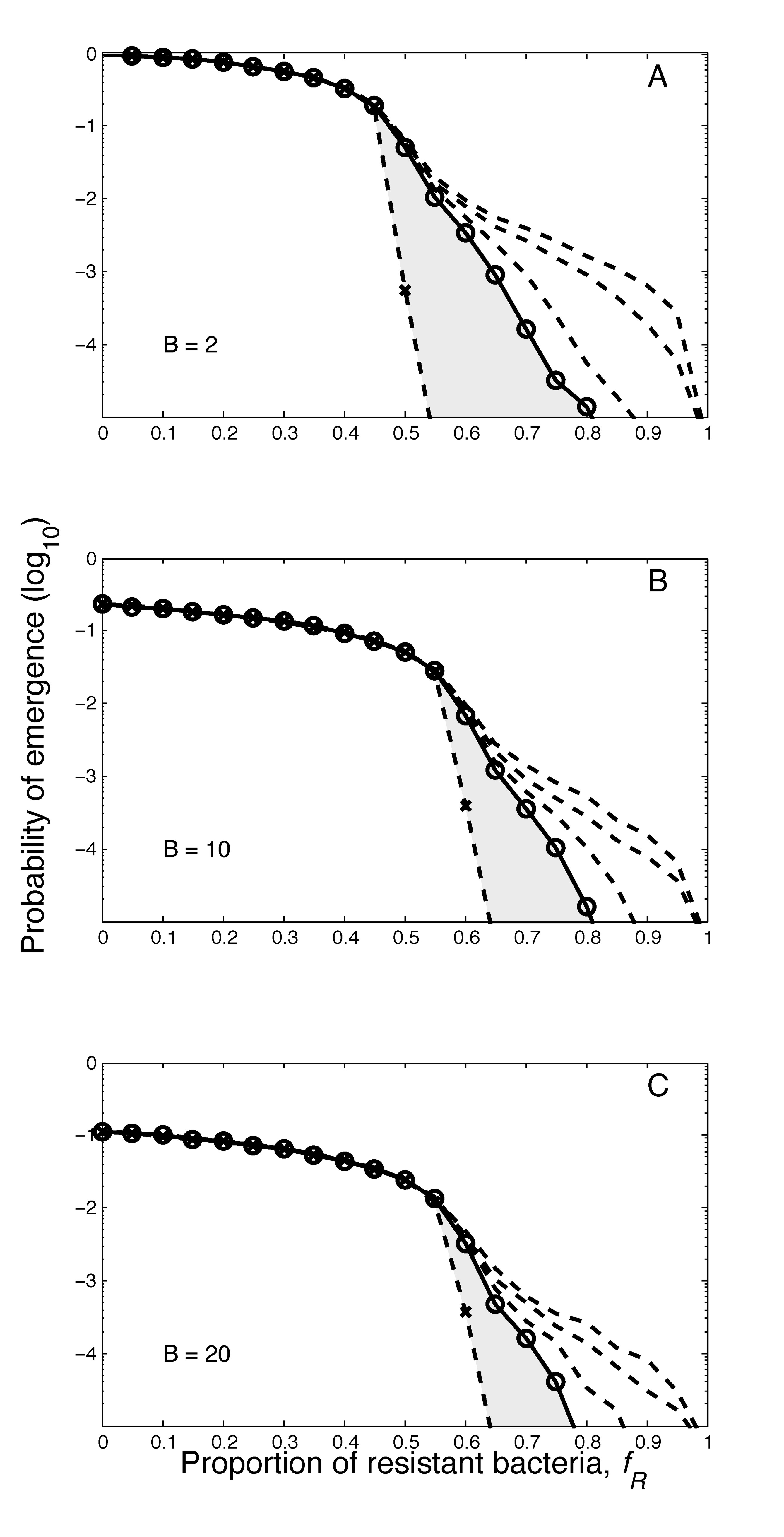

Supplement: S7 Fig — Results are shown on a log scale in the absence of mutation (u0,1 = 0, crosses, dotted line) or in the presence of mutation (u0,1 = 0.005), with one to four types of resistance (n = 1, 2, 3, dashed lines; n = 4, circles, solid line) for burst sizes B = 2, 10, and 20. Other parameter values: b^=2.5, d = 1, c = 0.05. Results are shown for 100,000 simulation runs. (TIF) [file pbio.2006738.s008.tif]

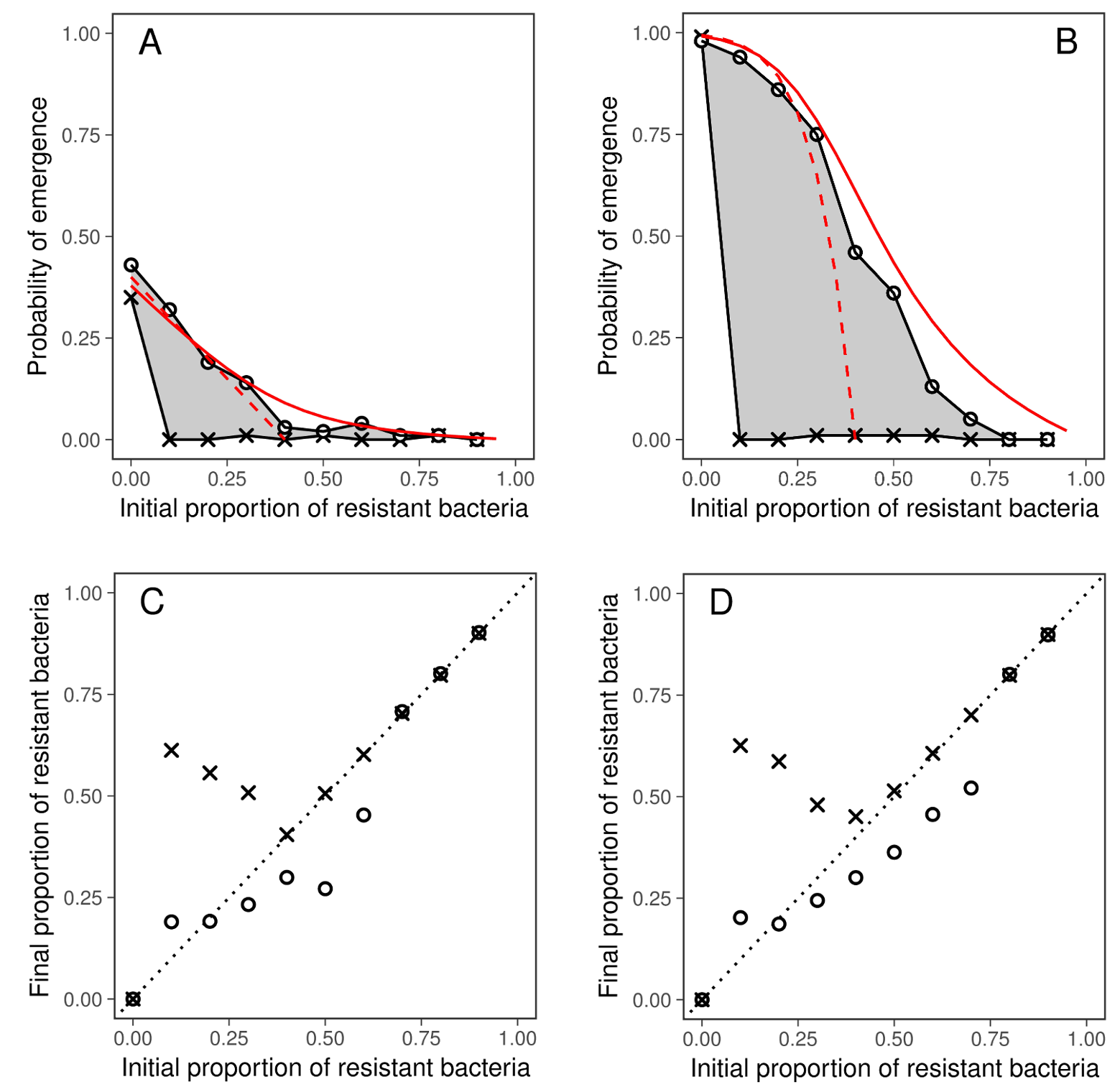

Supplement: S8 Fig — Upper panels (A and B): The probability of emergence versus the initial proportion of resistant bacteria in a well-mixed population (ϕ = 0) with a single type of resistance (n = 1). Results are shown in the absence (u0,1 = 0, crosses) or presence (u0,1 = 0.1, circles) of mutation, for initial inoculum sizes V0 = 1 (A) or V0 = 10 (B). For comparison, the predictions of the branching process approximation are shown (red lines). Lower panels (C and D): the fraction of resistant bacteria among uninfected bacteria at the end of the simulation for V0 = 1 (C) or V0 = 10 (D). Note the deviation from the y = x (dotted) line showing that the frequency of resistance is not constant. Other parameter values: b = 2.5, d = 1, c = 0.2. Results are shown for 100 simulation runs. Each simulation was stopped either when the parasite went extinct or when the maximal simulation time was reached (t = 200), depending on which event happened first. (TIF) [file pbio.2006738.s009.tif]

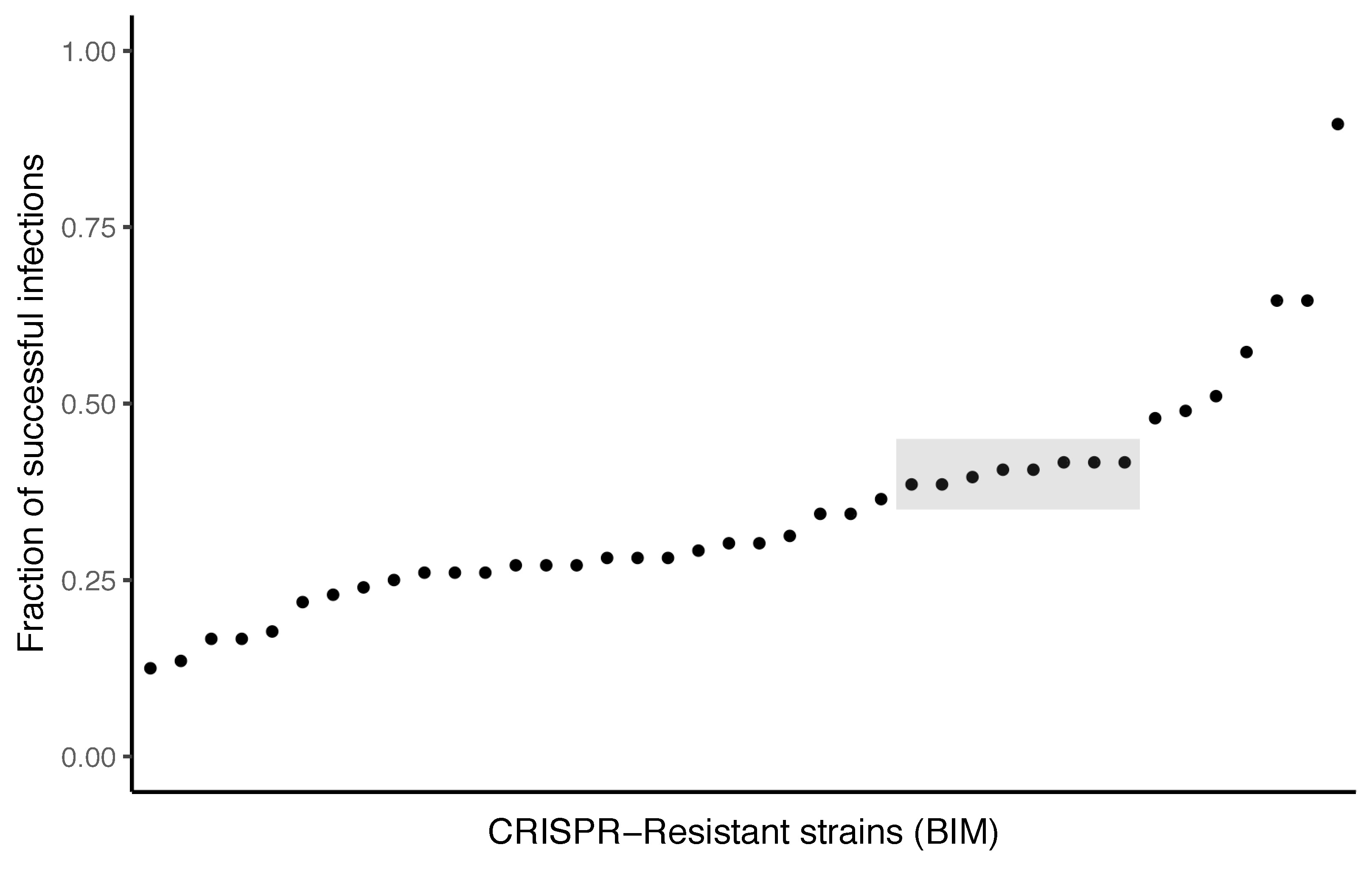

Supplement: S9 Fig — In Figs 4 and 5, we used eight different BIMs (indicated in the gray rectangle, see S2 Table), on which our estimation of the phage mutation rate was based (see section S2.1.6 of S1 Text). Data are available in S1 Data. BIM, bacteriophage-insensitive mutant. (TIF) [file pbio.2006738.s010.tif]

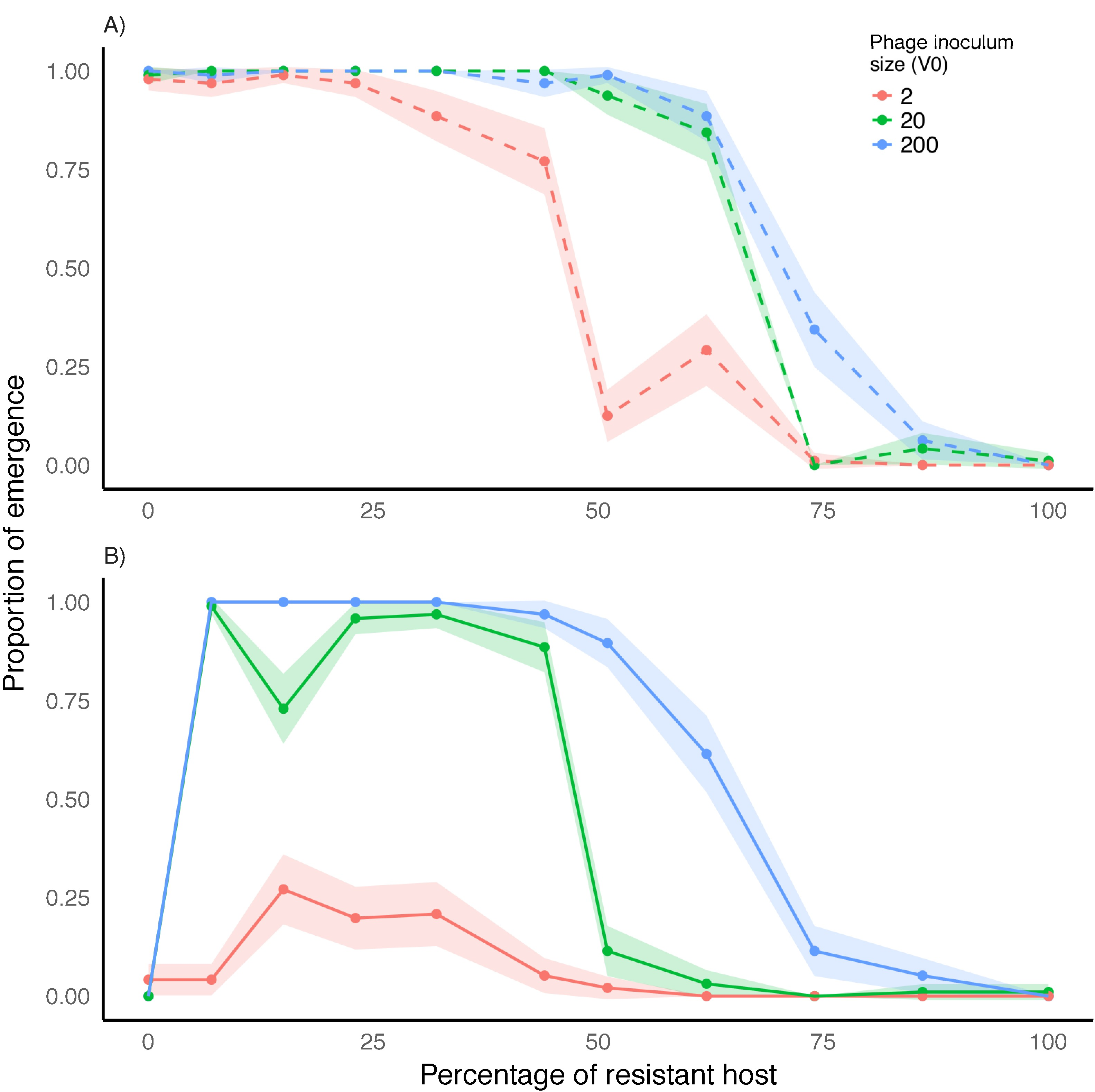

Supplement: S10 Fig — The proportion of populations (96 total) infected with either V0 ≈ 2 (red), V0 ≈ 20 (green), or V0 ≈ 200 (blue) phages, in which emergence (A, solid lines) or evolutionary emergence (B, dashed lines) was observed. Shaded areas represent estimated 95% confidence intervals. Data are available in S1 Data. (TIF) [file pbio.2006738.s011.tif]

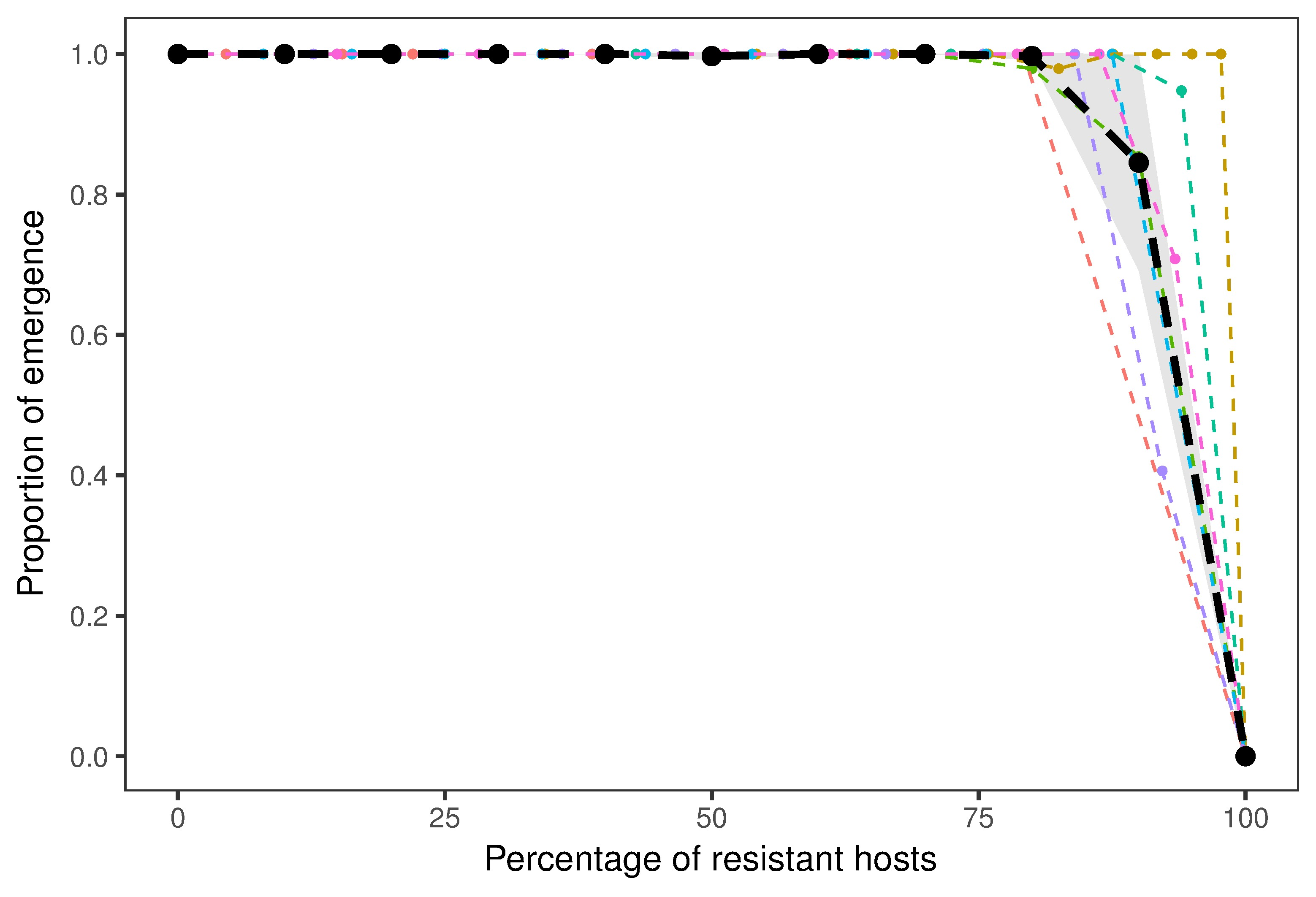

Supplement: S11 Fig — Probability of emergence (i.e., when the amplification of the phage is detected) for increasing values of the proportion of a single resistant bacterium (fR). In contrast, Fig 4 presents the probability of evolutionary emergence (i.e., when the amplification of an escape phage is detected). The different colors correspond to replicate experiments performed using eight different BIMs. For each treatment, each of the 96 replicate host populations were inoculated with an initial quantity of V0 ≈ 300 unevolved phages. Black lines indicate the mean across the eight BIMs; gray shaded areas represent 95% confidence intervals of the mean. Data are available in S1 Data. BIM, bacteriophage-insensitive mutant. (TIF) [file pbio.2006738.s012.tif]

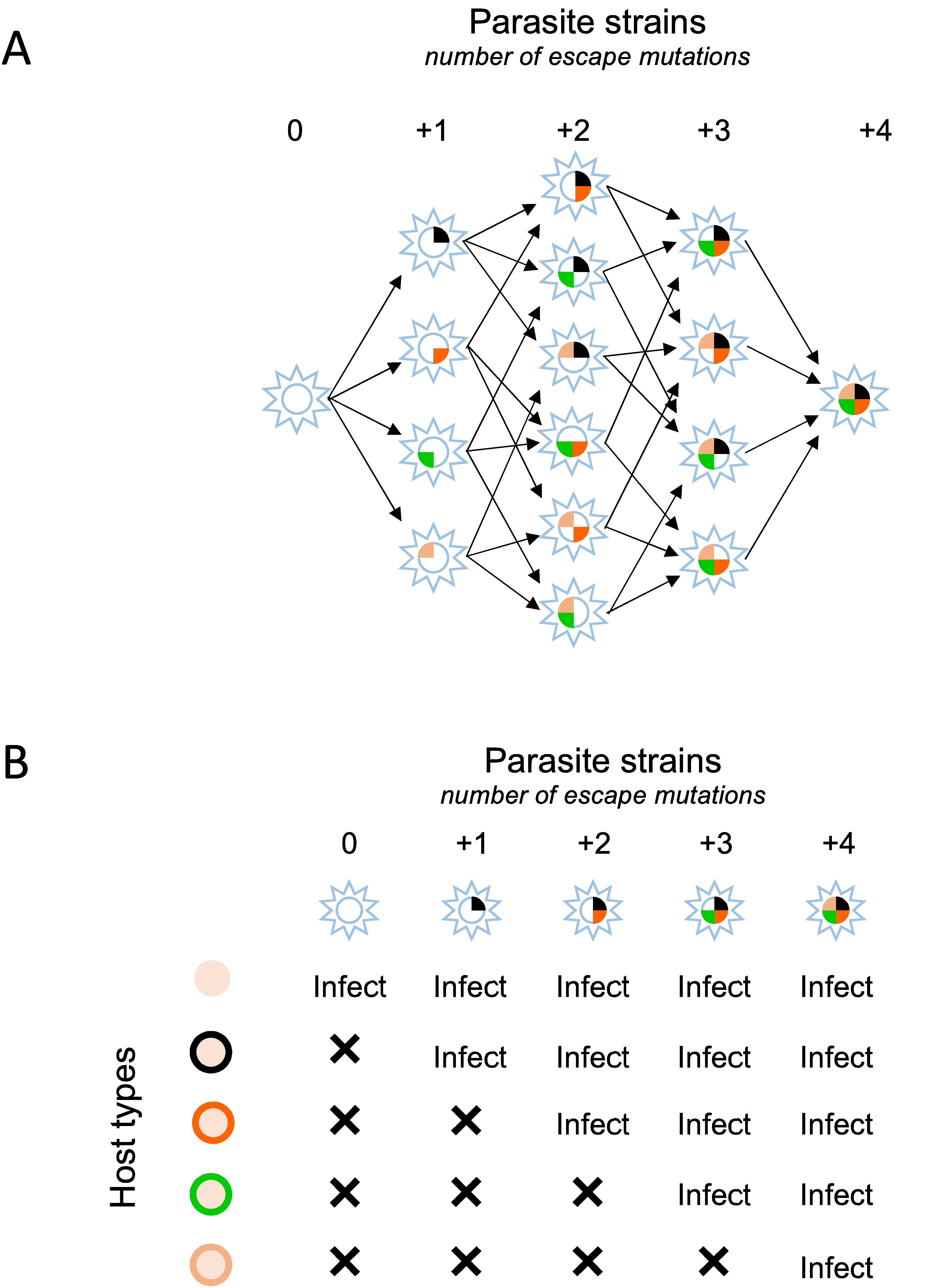

Supplement: S12 Fig — The figure (A) presents the network of 16 different strains with an increasing number of escape mutations. Arrows indicate possible mutation steps. The figure (B) presents the infectivity of five of these 16 different strains against each of the five different bacterial types (where a cross indicates bacteria resistance). (TIF) [file pbio.2006738.s013.tif]
