# Supplementary material for: Evolutionary emergence of infectious diseases in heterogeneous host populations
Source: PLoS Biol. 2018 Sep 24;16(9):e2006738. doi: 10.1371/journal.pbio.2006738 (PMC6171948; doi:10.1371/journal.pbio.2006738)
Supplement: S1 Table — (DOCX) [file pbio.2006738.s014.docx]

| **Main parameters** | **Definitions** |
| --- | --- |
| $b$ | Maximal transmission rate of the pathogen |
| $d$ | Death rate of infected hosts |
| $R_{0}=\frac{b}{d}$ | Maximal value of the basic reproduction ratio: when a pathogen with no escape mutations is spreading in a fully susceptible host population |
| $i$ | Number of escape mutations in a pathogen |
| $c$ | Cost of escape mutations on pathogen transmission rate |
| $b_{i}=b\left( 1-c \right)^{i}$ | Transmission rate of a pathogen with $i$ escape mutations |
| $f_{R}$ | Frequency of resistant hosts in the population |
| $n$ | Number of different resistant types in the host population (all resistant types are assumed to be equifrequent) |
| $\rho$ | Efficacy of immunity (from $0$ to $1$) |
| $\phi$ | Spatial structure of the population (from 0 to 1) |
| $F_{i,n}$ | Probability to infect a susceptible host for a free pathogen carrying $i$ escape mutations in a host population with $n$ types of resistant hosts |
| $R_{i,n}=\frac{b_{i}}{d}F_{i,n}$ | Basic reproduction ratio of a pathogen carrying $i$ escape mutations in a host population with $n$ types of resistant hosts |
| $\mu$ | Mutation rate of the pathogen per locus |
| $N$ | Genome size of the pathogen (number of loci) |
| $u_{i,n}=\mu\left( n-i \right)/\left( N-i \right)$ | Rate of acquisition of a new escape mutation of a pathogen which carries already $i$ escape mutations |
| $P_{i,n}=1-Q_{i,n}$ | Probability of emergence of of a single free pathogen with $i$ escape mutations in a host population with $n$ types of resistant hosts |
| $\hat{b}=b/\left( B-1 \right)$ | Transmission rate used in the burst-death model to obtain equivalent probability of emergence in the birth-death model |
