# Supplementary material for: Evolutionary emergence of infectious diseases in heterogeneous host populations
Source: PLoS Biol. 2018 Sep 24;16(9):e2006738. doi: 10.1371/journal.pbio.2006738 (PMC6171948; doi:10.1371/journal.pbio.2006738)
Supplement: S2 Table — BIM, bacteriophage-insensitive mutant. (DOCX) [file pbio.2006738.s015.docx]

| **BIM Name** | **BIM Spacer sequence (5’-3’)** |
| --- | --- |
| BIM 1 | CCGAACGCATANANGGCGCANGGCACAGGGGT |
| BIM 2 | TGAAAGTGCTGCTGGGTCTTCGCAGCCGGCAA |
| BIM 3 | GGCTCGACCAGGCGGCCCAGGGCGGCGTCGAT |
| BIM 4 | GCGGCAGGAGCGGCAGCGGGCGGCGGCAGTT |
| BIM 5 | AAGGGCATCAACCTGGCCGAAGGCGGCGCGCC |
| BIM 6 | GATGTTCATCGCTGCCGGGCAGCGCGACATAC |
| BIM 7 | AAACAGCGTCATGTCCAGGAGCTGCCGCTCGC |
| BIM 8 | CGCTGCCAGGCTGATGTCCTGGTTCAGCTCCA |
